# Two-sided matching based on I-BTM and LSGDM applied to high-level overseas talent and job fit problems

**DOI:** 10.1038/s41598-021-92057-7

**Published:** 2021-06-16

**Authors:** Qing Yang, Xinshang You, Yiye Zhang

**Affiliations:** 1 School of Accounting, Shanxi University of Finance and Economics, Taiyuan, 030006 China; 2 School of Economics and Management, Hebei University of Science and Technology, Shijiazhuang, 050018 China; 3 College of Law, Civil Aviation University of China, Tianjin, 300300 China

**Keywords:** Applied mathematics, Computer science

## Abstract

With the increasing number of overseas talent tasks in China, overseas talent and job fit are significant issues that aim to improve the utilization of this key human resource. Many studies based on fuzzy sets have been conducted on this topic. Among the many fuzzy set methods, intuitionistic fuzzy sets are usually utilized to express and handle the evaluation information. In recent years, various intuitionistic fuzzy decision-making methods have been rapidly developed and used to solve evaluation problems, but none of them can be used to solve the person-job fit problem with intuitionistic best-worst method (BWM) and TOPSIS methods considering large-scale group decision making (LSGDM) and evaluator social network relations (SNRs). Therefore, to solve problems of intuitionistic fuzzy information analysis and the LSGDM for high-level overseas talent and job fit, we construct a new hybrid two-sided matching method named I-BTM and an LSGDM method considering SNRs. On the one hand, to express the decision-making information more objectively and reasonably, we combine the BWM and TOPSIS in an intuitionistic environment. Additionally, we develop the LSGDM with optimized computer algorithms, where the evaluators’ attitudes are expressed by hesitant fuzzy language. Finally, we build a model of high-level overseas talent and job fit and establish a mutual criteria system that is applied to a case study to illustrate the efficiency and reasonableness of the model.

## Introduction

High-level overseas talent, which can be considered a part of natural globalization, has both advanced knowledge and international vision^1^. Such talent is an important force in Chinese economics, society, and development. To achieve the Chinese Dream, the Chinese government has paid close attention to the introduction and management of overseas talent, proposing that proper high-level overseas talent should be treated as an important force. There is a problem, which should not be ignored. The introduction of high-level overseas talent in China is largely driven by government policies, but the original initiative of both the supply and demand of human resources was limited. Although many overseas workers have great enthusiasm for coming to China, their knowledge and ability have not been fully utilized. Person-job fit is the key point to effectively achieve human resource allocation and management. Therefore, we need to underline the need for person-job fit in the process of overseas talent introduction and utilization. The two sides of person-job fit should take preferences and benefits into consideration. It is better to consider the two sides’ satisfaction degrees to improve the whole decision-making process effectiveness. To achieve this goal, we propose an optimization algorithm whose goal is to obtain the best matching degree of the person and job. The two-sided matching method is simply an appropriate method that considers the preferences of the two sides at the same time. In 1962, Gale and Shapley^2^ first proposed two-sided theory to solve the marriage problem. Since then, many researchers have shown great interest in this topic, extending this theory with different fuzzy language to different problems^3–5^. Some papers have used two-sided matching methodologies to solve human resource management problems^6–9^. Yu and Xu^10^ proposed a novel intuitionistic fuzzy two-sided matching model for the person-job problem. Compared with the mentioned papers, this paper discusses the Person-job fit problem for High-level overseas talent, which needs a systematic evaluation standard system determined by a large number of decision makers. With the development of management practice, the classical two-sided matching method is not sufficient. In particular, we need to find a proper description tool to express each decision body’s preference attitude. In what follows, we will introduce the best-worst method (BWM), the technique for order preference by similarity to ideal solution (TOPSIS) method and the large-scale group decision-making (LSGDM) method.

Additionally, an increasing number of academic studies have generally accepted that person-job fit should be characterized as the adaptability of characteristics and the compatibility between personnel and organizations. However, the interpretation of adaptation is not the same. There are three views explaining the connotation of person-job fit from different angles. The first is the requirements and capabilities view^11,12^, which considers that adaptation refers to the correspondence between job requirements and personal capability. The second is the similar or consistent view^13^, which believes that the person-job fit is due to certain similarities and mutual attractions, such as highly consistent values. Many scholars tend to agree with the third view of demand and supply^14^, under which people will choose organizations that have similar goals or can help them achieve their goals. Overall, regardless of the kind of view, it is essentially stressed that person-job fit is a mutual evaluation based on a specific criterion set. Therefore, a scientific criteria system is necessary. Due to the complexity of the evaluation standard system itself, this paper introduces a large-scale decision group to discuss the evaluation criteria and criteria weights. The traditional group evaluation problem will be difficult to utilize. Because most decision makers come from the same human resource management field, their relationship has a considerable impact on the weight of the evaluation criteria. In recent years, a large group decision-making method considering social networks (SN-LSGDM) has taken the social relationships of decision makers into account, bringing decisions closer to reality. For example, some decision makers may occupy important positions in the network structure. Their decision results may have a greater influence on the implementation of the decision results. Chu et al.^15^ took the reputation of decision makers into consideration, dealing with a group decision-making problem based on fuzzy preference relations. In particular, some papers^16,17^ have proposed the concept of leadership in social network analysis. Specifically, we let nodes represent the decision makers. If there is a relationship between two nodes, then we connect them. Some scholars have studied simple graphs, whose edges do not have directions and weights. Wu utilized the Louvain method to detect communities and calculated node weights by their degree centrality and eigenvector centrality^18^. Chu et al.^15^ discussed decision makers’ social relations using directed graphs, which are more complicated and closer to reality. Wu et al.^19^ studied a linguistic relation with incomplete information. In reference^18^, Furthermore, this paper considers the importance of nodes and the influence of modularity on the overall structure of a network.

## Literature review

### Best-worst-method (BWM)

The BWM is a different idea for ranking alternatives than the pairwise comparison methods proposed by Rezaei^20^ in 2015, applying to a phone choosing problem. Then he^21^ applied a linear BWM to a car choosing problem, which used several properties of the BWM. One highlight of the BWM is that the pairwise comparison time drops from $n(n-1)/2$ to $2n-3$. The BWM has attracted much attention from scholars^22^ in different fields. Liang^23^ used linguistic variables and fuzzy numbers from a qualitative and quantitative perspective to not only enrich the expression of decision-makers but also make their opinions very direct and effective. Guo and Zhao^24^ extended the BWM to fuzzy environments. Yang et al.^25^ extended the BWM to the I-BWM by introducing intuitionistic preference relations. Ahmadi et al.^26^ studied social sustainability importance in supply chains using BWM by involving 38 experts. Kheybari et al.^27^ used the BWM with more than 40 experts to determine the weight of energy and locational factors for the location selection problem. Some scholars have extended the BWM to a mixed method, such as BWM and VIKOR^23^. As the idea of the BWM decreases the number of comparisons, the BWM will not be suitable when the number of decision makers is large and their relationships are complex. In this paper, the BWM will be discussed under the environment of preference relations.

In general, the BWM has 3 main steps:

Step 1: Determine the best and worst criteria from among *n* alternatives.
Step 2: Determine the preference intensity degree of the best alternative over than the others except for the worst one, which requires $n-2$ comparisons. The remaining $n-2$ alternatives need to be compared to the worst one, which requires $n-2$ comparisons. Adding the comparisons of the best alternative to the worst, we need $(n-2)+(n-2)+1=2n-3$ times of comparisons in all.
Step 3: Calculate the weights of all alternatives and rank them.

The result of Best-to-Others vector is: $A_{B}=(a_{B1},a_{B2},\ldots ,a_{Bn})^{T}$; the result of Others-to-Worst vector is: $A_{W}=(a_{1W},a_{2W},\ldots ,a_{nW})^{T}$. The optimal weight for the criteria is the one where, for each pair of $w_{B}/w_{j}$ and $w_{j}/w_{W}$, we have $w_{B}/w_{j}=a_{Bj}$ and $w_{j}/w_{W}=a_{jW}$ for all *j* is minimized. Considering the non-negativity of weights, we obtain:

$$min\; \; max\left\{\left|\frac{w_{B}}{w_{j}}-a_{Bj}|,|\frac{w_{j}}{w_{W}}-a_{jW}\right|\right\}$$

s.t.

$$\sum w_{j}=1, \; w_{j}\ge 0 \; \text{for} \; \text{all} \; j.$$

The optimal weights $(w_{1}^{*},w_{2}^{*},\ldots ,w_{n}^{*})$ could be calculated by

$$min \xi$$

s.t.

1 $$\begin{aligned} \left\{ \begin{array}{l} |\frac{w_{B}}{w_{j}}-\alpha _{x_{Bj}}|\le \xi ,\\ |\frac{w_{j}}{w_{W}}-\alpha _{x_{jW}}|\le \xi ,\\ w_{k}\ge 0, \forall j,\\ \sum w_{j}=1. \end{array} \right. \end{aligned}$$

where $$j=1,2,\ldots ,n$$.

As noted for the BWM method, it is not difficult for people to choose the best and the worst among the alternatives under a certain criterion. Determining by how much the best alternative is superior to the others and by how much the others are superior to the worst are the difficult steps. The BWM is an effective method for dealing with comparison times. Moreover, the BWM expresses the comparison results with numbers in the set $\{1, 2, 3, 4, 5, 6, 7, 8, 9\}$ and ignores the reciprocals of each pair to avoid the difficulties caused by unequal distances between fractional comparisons. In this paper, the decision body expresses its preference attitude with the I-BWM. We introduce a transformation formula to obtain the corresponding intuitionistic fuzzy numbers, aggregating the initial evaluation results more effectively.

### TOPSIS method

**Figure 1:**
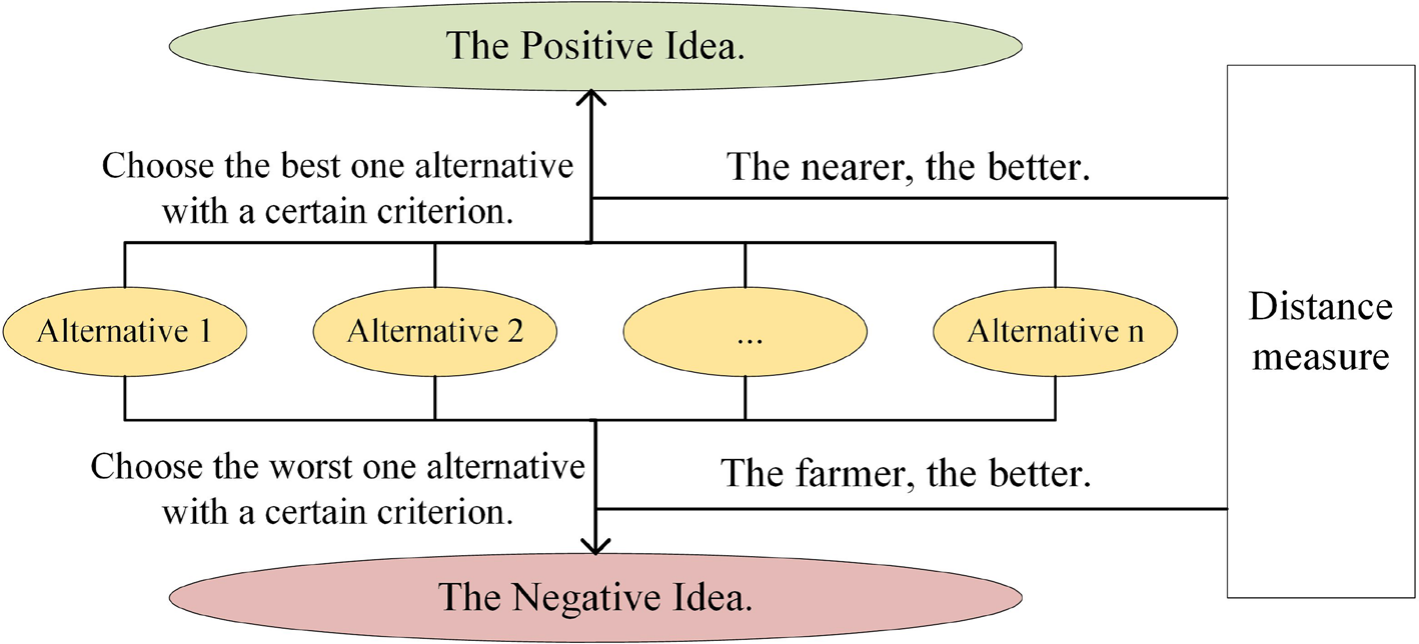
Evaluation idea of Topsis method.

To handle the satisfactory weight calculation problem, we introduce the technique for order preference by similarity to ideal solution (TOPSIS) method to calculate satisfactory weights. TOPSIS is a well-known MCDM method, proposed by Huang and Yang^28^ in 1981 and studied by many researchers, policymakers and stakeholders, who have mostly aimed to promote and improve the core functions of this method^29–31^. The core idea of the TOPSIS method is that the closer to the positive ideal solution and the farther from the negative ideal solution an alternative is, the better it is, as shown in Fig. 1.

Due to its reasonable logic and ease of understanding, TOPSIS has become famous and has been widely applied to address the MCDM problem^32^. Ye^33^ used it to solve the partner choosing problem, which depends on interval-valued intuitionistic fuzzy sets. Wang et al.^34^ combined the TOPSIS method with the ordered weighted averaging (OWA). Joshi et al.^35^ ranked the alternatives by the TOPSIS method, considering a distance measure under the intuitionistic environment. Paritosh et al.^36^ applied the TOPSIS method to select the best possible alternative in solid-state anaerobic digestion. Opricovic et al.^37^ gave the correlation coefficient formula of the TOPSIS method between two distances. Then, Kuo^38^ constructed a ranking method for both of the positive and negative ideal distances. These papers made some contributions to the TOPSIS method system. Some authors have studied this method in different situations, such as using type-2 fuzzy numbers^18^ and intuitionistic fuzzy numbers^39^.

Since Huang and Yoon first introduced TOPSIS, it has been a popular MCDM method. The main idea of TOPSIS is to find the best alternative that has the greatest distance from the negative ideal solution and the least distance from the positive solution. Measuring distance is a complex problem that has been researched by many scholars. Generally, TOPSIS has the following steps:

Step 1: Determine the evaluation results for the alternatives considering each criterion.
Step 2: Determine the weight vector of the criteria.
Step 3: Determine the positive and negative ideal solutions.
Step 4: Determine the distance measure to calculate each alternative’s ratio, which is obtained by considering the distance from the positive ideal and the distance from the negative ideal.
Step 5: Rank the alternatives according to their ratios.

Thus far, the mentioned papers have not been suitable for large-scale group decision-making problems. Works in this field are still related to traditional decision-making methods. In our study, the extended TOPSIS method is improved on the basis of multiobjective optimization and is then used to identify the optimal design scheme. Overall, compared with the traditional TOPSIS methods, the merits of the proposed new TOPSIS method are threefold: (1) a nonlinear optimization BWM model to evaluate talent is constructed based on the TOPSIS method’s comparison idea; (2) the proposed TOPSIS method depends on a social analysis process, which determines the weights of the decision makers and criteria from both the social relations and decision information of the decision makers; and (3) based on the TOPSIS method, the problem of person-job matching is further improved. Distance measurement is a key aspect of the TOPSIS method. We propose a novel distance measure to calculate the distance between different intuitionistic fuzzy numbers.

### Large-scale group decision making (LSGDM) method

When the number of decision makers is more than 20, the traditional multicriteria decision-making methods are invalid. This issue is called the large-scale group decision-making (LSGDM) problem. There is a trend in which a larger number of experts are becoming involved in the decision-making process. Therefore, the large-scale group decision-making problem has become a much-discussed topic^40^. Many challenges stemming from general GDM and LSGDM have arisen. Generally, there are two aspects of these challenges: the consensus degree of the decision results and the decision makers’ social network relations, which are important for determining the decision makers’ and criteria’s weights. Some researchers have focused on the consensus reaching process, which aims to bring the DMs’ preferences closer through rounds of discussions, negotiations and communications. Tang et al.^40^ overcame the limitation of the subgroup size, determining the subgroup weight by its members’ conflict degree. However, decision makers’ social network relations were not taken into consideration. Decision makers’ complex social relations will affect their decision results to some extent. It is necessary to consider the decision results with their connections. Ding et al.^41^ also studied the clustering method for large-group decision making models, summarizing the related methods. From Ding’s paper^41^, the decision makers’ weights are determined by two kinds of criteria about their importance to their social network. In this paper, we also weight the criteria and decision makers by their importance degree. In addition, we study the decision makers’ connections within subgroups and their influence on the other subgroups. On the one hand, our clustering method makes the subgroups’ weight values more objective. On the other hand, our distance measures based on the BWM are more suitable for evaluating problems.

**Figure 2:**
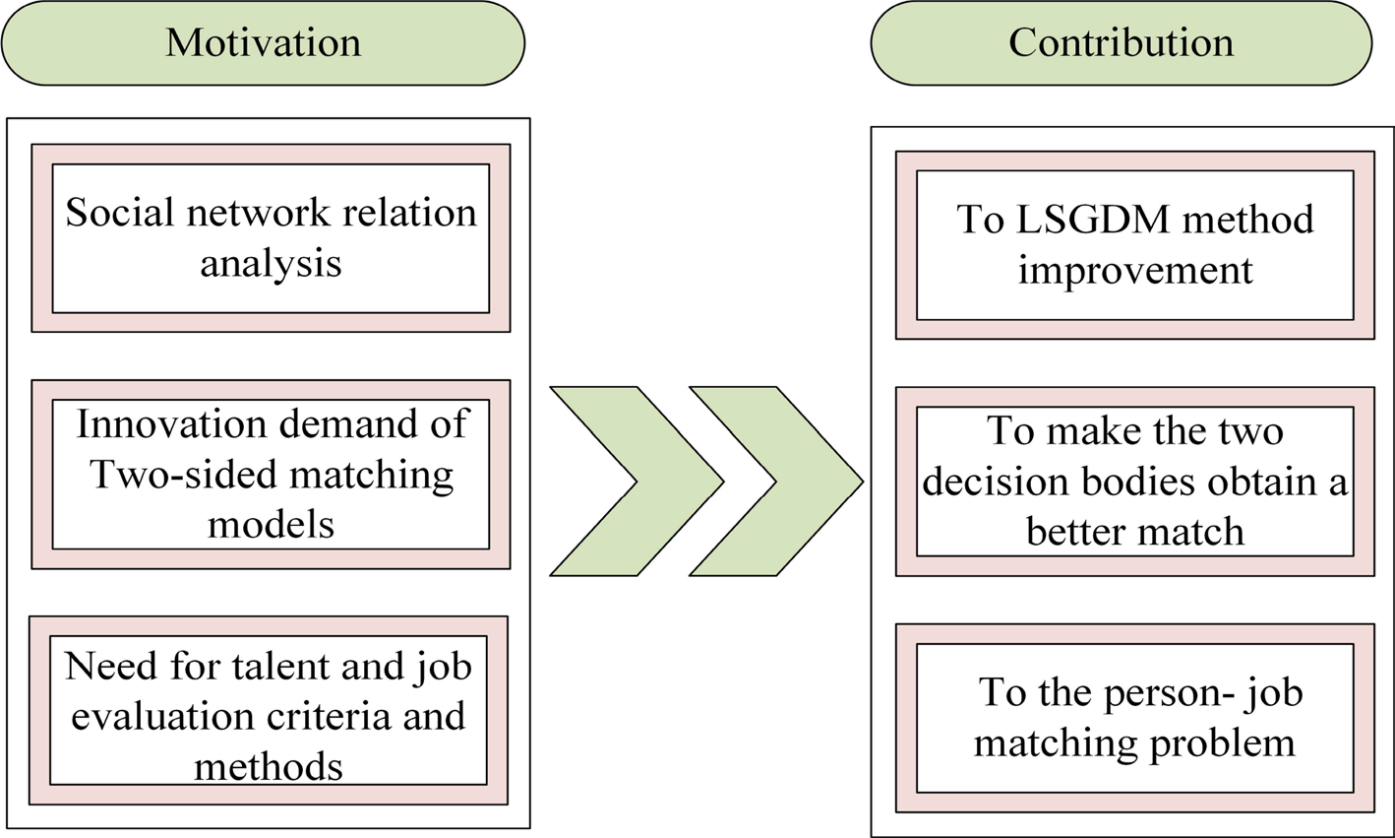
Motivations of this paper.

The motivations of this paper are summarized in three parts, as shown in Fig. 2. China regards talent as the primary driving force of innovation, and talent evaluation covers a wide range. Additionally, the number of decision bodies for the person-job matching problem is larger than that for a traditional evaluation problem. With the increase in the number of decision-makers, the impact of their social network relationship on the determination of evaluation results is becoming increasingly prominent. This paper weights the evaluation criteria by analyzing the decision makers’ social network relationships. The proposed clustering algorithm based on social network analysis is helpful in enriching and improving the LSGDM problem system. For the person-job fit problem, it is of great significance to evaluate the efficient matching of both decision bodies. Therefore, the two-sided matching model proposed in this paper will enrich the research method system of this problem. To carry out the evaluation process more smoothly, this paper systematically constructs an evaluation standard system covering both sides and applies it to solve practical problems. In the process of constructing the methodology, some innovative definitions and theorems are given to support the methodology.

## Network analysis in the large-scale group decision making (LSGDM) problem

In this section, we introduce the hesitation degree of probabilistic linguistic terms (PLTs) to describe decision makers’ evaluations of the LSGDM problem^42^. Pang et al.^43^ put forward the concept of probabilistic linguistic term set (PLTS), which allows decision makers (DMs) to choose several linguistic terms from a linguistic term set (LTS) and associate them with the probabilities so as to express their information more accurately. To address large-scale numbers, large-scale group decision makers will be assigned to subgroups by applying Algorithm 1 below. Then, the subgroups’ weights will be calculated by Algorithm 2.

### Basic definitions for LSGDM

LTS can be also named linguistic evaluation scale, which consists of an odd number of ordered linguistic terms^44^. It determines the range of linguistic terms, which are available for the linguistic computational models. One commonly LTSs is $S=\{S_{\alpha }|\alpha =-\tau ,\ldots ,-1,0,1,\ldots ,\tau \}$, where $\tau$ is a positive number. Based on the subscript-symmetric LTS, the concept of PLTSs is shown below. During the decision-making process, although $2\tau +1$ evaluation grades are proposed to choose, DMs have different backgrounds and expertise levels, leading to different hesitation degrees. On the one hand, the hesitation degree reflects the decision makers’ preference degrees. On the other hand, it also represents the criterion’s distinction degree, where the higher the hesitation degree is, the lower the weights obtained. To measure an evaluation result’s hesitation degree, the following definition is given:

^43^ Let $L_{P}=\{L^{k}(p^{k})|L^{k}\in S, p^{k}\ge 0, k=1,2,...,\sharp L(P)\}$ be a set of PLTs, $k\in \{-\tau ,\ldots ,0,\ldots ,\tau \}$. $P_{L}=\{(p^{k})|\Sigma ^{P_{L}}_{k=1}\le 1\}$ is a set of probabilistic from PLTs, and $\sharp H(P)$ is the number of linguistic terms. The hesitant degree function *Hd*(*P*) of $L_{P}$ is defined as below.

2 $$\begin{aligned} Hd(L_{P})=\frac{\sharp L_{P}^{2}-1}{(2\tau +1)^{2}-1}\times \frac{1}{\sum P_{L}} \end{aligned},$$

where the probabilistic degree should be multiple of $\frac{1}{2\tau +1}$.

Meanwhile, each decision result’s mathematical expectation could be calculated by Eq. (3).

3 $$\begin{aligned} E(L_{P})=\sum ^{\sharp L(P)}_{k=1}p^{k}s^{k} \end{aligned},$$

$s^{k}$ standing for the level of the PLT. Such as a decision maker’s evaluation is $\{s_{-1}(\frac{1}{7}),s_{0}\left(\frac{3}{7}\right),s_{1}\}\left(\frac{2}{7}\right)$, then its mathematical expectation is $\frac{1}{7}\times (-1)+\frac{3}{7}\times 0+\frac{2}{7}\times 1=\frac{1}{7}.$

Then a decision maker’s final integrated decision result could be calculated by Eq. (4).

4 $$\begin{aligned} F(L_{P})=E(L_{P}) \times Hd(L_{P}) \end{aligned}.$$

#### Theorem 1

*The function* $Hd(L_{P})$ *is bounded, a strictly monotonically increasing and concave function, where* $Hd(L_{P})$ *is about independent constant variable* *x*.

#### Proof

Because $0\le \sharp L_{P}\le 2\tau +1$, then $\frac{\sharp L_{P}^{2}-1}{(2\tau +1)^{2}-1\in [0,1]}$. And $0\le \sum P_{L}\le 1$, the probabilistic value is multiple of $\frac{1}{2\tau +1}$, then $\frac{1}{\sum P_{L}}\in [1,2\tau +1]$, which is bounded. Thus, function $Hd(L_{P})$ is bounded. The first derivation of $Hd(L_{P})$ is $\frac{d(Hd(L_{P}))}{dx}=\frac{1}{\{(2\tau +1)^{2}-1\}\sum P_{L}}\times 2x$, where $0\le 2x\le 2\tau +1$, then $\frac{d(Hd(L_{P}))}{dx}\in [0,1]$. Therefore, the function $Hd(L_{P})$ is a strictly monotonically increasing. In addition to calculated the second derivation of $Hd(L_{P})$, obtaining $\frac{d^{2}(Hd(L_{P}))}{dx^{2}}=\frac{2}{\{(2\tau +1)^{2}-1\}\sum P_{L}}$, demonstrates the function $Hd(L_{P})$ is concave function. $\square$

### Aggregating algorithm constructed with network analysis

An important definition in this section is modularity, which is used to address aggregation problems and is defined by Eq. (5).

5 $$\begin{aligned} \Delta Q=\left[ \frac{W_{c}+s_{i,in}}{2W}-\left( \frac{S_{c}+s_{i}}{2W}\right) ^{2}\right] -\left[ \frac{W_{c}}{2W}-\left( \frac{S_{c}}{2W}\right) ^{2}-\left( \frac{S_{i}}{2W}\right) ^{2}\right] \end{aligned},$$

where $s_{i,in}$ is the edge number of node *i* and the other nodes in community *C*.

A principle of network analysis in the LSGDM problem is to let each node be a separate community initially. To maximize the modularity of the whole community, we calculate the largest local contribution of each node community. The basic algorithm process contains four steps.

**Algorithm 1**

Step 1: Let each node to be an original community.
Step 2: Based on the modularity, some neighbors are determined to be merged, after one iteration.
Step 3: Each community is regarded as a new node. We calculate its degree and connection information, which is the basis for the next iteration.
Step 4: Repeat step 3 until the modularity of the community no longer increases.

### A method for calculating the subgroups’ weight vectors

The aggregation results are calculated by the fast greedy method based on the modularity gain degree. In reference^18^, Wu et al. used the Louvain method^45^ to analysis the decision maker’s social network relations depending on the modularity. Inspired by Wu’s aggregating method, we utilize the fast greedy method to analysis the decision makers’ relationships. The fast greedy method’s core idea was proposed by Newman^46^. Different with Wu’s method, we measure the degree of intimacy based on the whole network structure. Zhang et al.^47^ used this measure to deal with the LSGDM problem during evaluating collaborative innovation degree. To prevent dense connections inside a group and sparse connections to the outside, we determine the subgroups’ weight vectors based on two aspects. One aspect is the node degree and eigenvector centralities; the other aspect is the influence of each subgroup on the others. In a subgroup, a node has two kinds of edges, an inside edge and an outside edge. The inside edge connects nodes belonging to the same subgroup. The outside edge connects two kinds of nodes: one belongs to the subgroup, and the other belongs to the outside. A subgroup may receive high weight for the nodes contained in this subgroup with high degrees. However, the connections of this subgroup and the outside nodes may not be as dense as the inside. Depending on the aggregation method, all *T* nodes will be separated into *S* parts, and each subgroup will have $Q_{s}$ nodes, $s\in \{1,2,\ldots ,S\}$. The main processes for determining the subgroups’ weights are given below.

**Algorithm 2**

Step 1: Calculate each node’s degree centrality (DC) and eigenvector centrality (EC). Combine the two centralities to obtain a node weight $\lambda _{node,t}$ for node *t*, by Eq. (6), and normalize .
  6 $$\begin{aligned} \lambda _{node,t}= & {} \sqrt{DC_{t}\cdot EC_{i}} \end{aligned},$$
  7 $$\begin{aligned} \overline{\lambda _{node,t}}= & {} \frac{\lambda _{node,t}}{\sum ^{T}_{t=1}\lambda _{node,t}} \end{aligned}.$$
  Based on all nodes weight, the network’s average centrality $\lambda _{net}$ is computed by Eq. (8) and the subgroup’s average centrality $\lambda ^{s}_{comm}$ is computed by Eq. (9):
  8 $$\begin{aligned} \lambda _{net}= & {} \frac{1}{T}\sum ^{T}_{t=1}\lambda _{node,t} \end{aligned},$$
  9 $$\begin{aligned} \lambda ^{s}_{comm}= & {} \frac{1}{S_{t}}\sum ^{S_{t}}_{t=1}\lambda _{node,t} \end{aligned}.$$
  A subgroup’s inside weigh $\lambda _{in,s}$ is determined by the distance of this subgroup’s average centrality and the network’s average centrality, computed by Eq. (10).
  10 $$\begin{aligned} \lambda _{in,s}=\frac{1}{\lambda ^{s}_{comm}-\lambda _{net}} \end{aligned}.$$
  And normalize $\lambda _{in,t}$ by Eq. (11), obtaining $\overline{\lambda _{in,s}}$.
  11 $$\begin{aligned} \overline{\lambda _{in,s}}=\frac{\lambda _{in,s}}{\sum ^{Q_{s}}_{s=1}\lambda _{in,s}} \end{aligned}.$$
Step 2: Suppose there are $Q_{s}$ nodes in subgroup $C_{s}$. Calculate the number of edges for all $Q_{s}$ nodes, which connects the inside nodes and outside nodes, denoted as $\lambda _{out,s}$. Normalize $\lambda _{out,s}$ and obtain $\overline{\lambda _{out,s}}$, an outside weight $\lambda _{out,s}$, with Eq. (12). $\alpha$ is a moderator variable that can adjust the importance degree of $\overline{\lambda _{in,s}}$ and $\overline{\lambda _{out,s}}$.
  12 $$\begin{aligned} \overline{\lambda _{out,s}}=\frac{\lambda _{out,s}}{\sum ^{S}_{s=1}\lambda _{out,s}} \end{aligned}$$
Step 3: Combine the inside weight and outside weight of the network together with Eq. (13), to obtain the subgroup’s weight $\lambda$:
  13 $$\begin{aligned} \lambda =\alpha \overline{\lambda _{in,s}}+(1-\alpha )\overline{\lambda _{out,s}} \end{aligned}.$$

## A novel two-sided decision making model

### Basic definitions

Let $X=\{x_{1},x_{2},\ldots ,x_{n}\}$ be a nonempty finite set with *n* elements^48^. Then, the intuitionistic multiplicative preference relations (IMPR) is defined as $A=(\alpha _{ij})_{n\times n}$, where $\alpha _{ij}=(\rho _{\alpha _{ij}},\sigma _{\alpha _{ij}})$ is called an intuitionistic multiplicative number (IMN) for all $i,j\in \{1,2,\ldots ,n\}$ and $\rho _{ij}$ indicates the intensity to which $x_{i}$ is preferred to $x_{j}$, $\sigma _{ij}$ indicates the intensity to which $x_{i}$ is not preferred to $x_{j}$, and both should satisfy the following conditions:

$$\rho _{\alpha _{ij}}=\sigma _{\alpha _{ji}},\; \rho _{\alpha _{ji}}=\sigma _{\alpha _{ij}}, \; \rho _{\alpha _{ii}}=\sigma _{\alpha _{ii}}=1, \; 0\le \rho _{\alpha _{ij}}\sigma _{\alpha _{ij}}\le 1,\; 1/9\le \rho _{\alpha _{ij}},\sigma _{\alpha _{ij}}\le 9.$$

In addition, let $\tau _{ij}=1/(\rho _{ij}\sigma _{ij})$, i.e. $\tau _{ij}\rho _{ij}\sigma _{ij}=1$. Here, $\tau _{ij}$ represents the hesitation degree to which $x_{i}$ is preferred to $x_{j}$, which satisfies $\tau _{ij}\in [1,81]$.

Let $X=\{x_{1},x_{2},\ldots ,x_{n}\}$ be a set of *n* alternatives^49^. An intuitionistic fuzzy set (IFS) is defined as $U=\{\langle x,u_{x},v_{x}\rangle |x\in X\}$, where $0\le u_{x}+v_{x}\le 1$, $0\le u_{x}, v_{x}\le 1$, and $u_{U}(x)$ is the membership degree of *x* to *X*, and $v_{U}(x)$ is the nonmembership degree of *x* to *X*. The hesitant degree of *x* to *X* is determined by $\pi =1-u_{x}-v_{x}$. An intuitionistic fuzzy number (IFN) is written as $(u_{x},v_{x})$, or (*u*, *v*) for simplicity.

 Let $X=\{x_{1},x_{2},\ldots ,x_{n}\}$ be a nonempty finite set with *n* elements^50^. Its associated multiplicative reciprocal preference relation $A=(a_{ij})$ with $a_{ij}\in [1/9,9]$ and $a_{ij}\cdot a_{ji}=1$, $\forall i,j\in \{1,2,\ldots ,n\}$. The corresponding fuzzy reciprocal preference relation associated with *A* is given as follows:

14 $$\begin{aligned} p_{ij}=f(a_{ij})=\frac{1}{2}(1+\log _{9}a_{ij}) \end{aligned},$$

where $$p_{ij}\in [0,1]$$ and $$p_{ij}+p_{ji}=1$$, $$\forall i,j\in \{1,2,\ldots ,n\}$$.

Depending on Eq. (14) and the definition to measure the distance between two IMNs^25^, we have the following transformation function *f* for any IMN $\alpha _{ij}=(\rho _{\alpha _{ij}},\sigma _{\alpha _{ij}})$:

15 $$\begin{aligned} t_{ij}=f(a_{ij})=(log_{9}\rho _{\alpha _{ij}},1+1og_{(1/9)}\sigma _{\alpha _{ij}}) \end{aligned}.$$

Let $X=\{x_{1},x_{2},\ldots ,x_{n}\}$ be a nonempty finite set with *n* elements^51^. Define the intuitionistic fuzzy set (IFS) $A=\{\langle a,\mu _{A}(x),\nu _{A}(x)\rangle |x\in X\}$ on *X* by the functions $\mu _{A}(x)$ and $\nu _{A}(x)$, which satisfy $0\le \mu _{A}(x),\nu _{A}(x)\le 1$, $0\le \mu _{A}(x)+\nu _{A}(x)\le 1$. The function $\pi _{A}(x)=1-\mu _{A}(x)-\nu _{A}(x)$ represents the hesitation degree of *a* to *A*.

Xu^52^ named $a=(\mu ,\nu )$ an intuitionistic fuzzy number (IFN) for simplicity.

### Best-worst-method (BWM) for intuitionistic relations

Let $X=\{x_{1},x_{2},\ldots ,x_{n}\}$ be a nonempty finite set with *n* alternatives, and let $C=\{c_{1},c_{2},\ldots ,c_{m}\}$ be a set with *m* criteria. Determine the best element $x_{B}$ and the worst element $x_{W}$ with respect to a certain criterion. Denote the comparison value $x_{B}$ over $x_{j}$ by $x_{Bj}$, $\forall x_{j}\in X$, in the form of an intuitionistic multiplicative number (IMN), to compose a set $S_{B}=\{x_{B1},\ldots ,x_{Bn}\}$ and elements in it called best-grade comparisons. Denote the comparison value $x_{i}$ over $x_{W}$ by $x_{iW}$, $\forall x_{i}\in X$, in the form of an IMN, composing a set $S_{W}=\{x_{1W},\ldots ,x_{nW}\}$ and elements in it are called the Worst-grade comparisons. Moreover, the elements of $S_{B}$ and $S_{W}$ are $x_{ij}=(\alpha _{x_{ij}},\beta _{x_{ij}})$, where $\alpha _{x_{ij}}$ is the preference degree of $x_{i}$ over $x_{j}$ expressed as an integer between 1 and 9 and $\beta _{ij}$ is the nonpreferred degree of $x_{i}$ over $x_{j}$, expressed by a number among $\{1,1/2,\ldots ,1/9\}$, and they satisfy the conditions that $\alpha _{x_{ij}}=\beta _{x_{ji}}$, $\beta _{x_{ij}}=\alpha _{x_{ji}}$ and $\alpha _{x_{ij}}\beta _{x_{ji}}\le 1$, which indicates that the hesitation degree is under consideration.

In this section, we introduce the optimization model of the BWM^53^ for intuitionistic preference relations. First of all, the decision maker should ensure the criteria set $C=\{C_{1},C_{2},\ldots ,C_{n}\}$, with respect to an alternative set $X=\{x_{1},x_{2},\ldots ,x_{n}\}$, which will be identified to yield pairwise comparisons. The best element $x_{B}$ is selected, as well as the worst element $x_{W}$. Then, decision makers enter the comparison results for $x_{B}$ compared with the others and the remaining elements compared with $x_{W}$, then we obtain comparison sets $S_{B}$ and $S_{W}$. Since the deviation results concern two aspects, the preferred degree and the nonpreferred degree, then we should consider the following problem:

16 $$\begin{aligned} min\left\{ \left| \frac{w_{B}}{w_{k}}-\alpha _{x_{Bk}}\right| ,\left| \frac{w_{k}}{w_{W}}-\alpha _{x_{kW}}\right| \right\} ; min\left\{ \left| \frac{w_{k}}{w_{B}}-\beta _{x_{Bk}}\right| ,\left| \frac{w_{W}}{w_{k}}-\beta _{x_{kW}}\right| \right\} \end{aligned},$$

where $$w_{k}\ge 0$$, $$\sum w_{k}=1$$, $$k=1,2,\ldots ,n$$.

We can deal with this problem by solving the following systems:

**Model 1**

*min* $$\xi$$ s.t.:

17 $$\begin{aligned} \left\{ \begin{array}{l} \left| \frac{w_{B}}{w_{k}}-\alpha _{x_{Bk}}\right| \le \xi ,\\ \left| \frac{w_{k}}{w_{W}}-\alpha _{x_{kW}}\right| \le \xi ,\\ w_{k}\ge 0,\\ \sum w_{k}=1. \end{array} \right. \end{aligned},$$

where $$k=1,2,\ldots ,n$$.

*min* $$\eta$$ s.t.:

18 $$\begin{aligned} \left\{ \begin{array}{l} \left| \frac{w_{k}}{w_{B}}-\beta _{x_{Bk}}\right| \le \eta ,\\ \left| \frac{w_{W}}{w_{k}}-\beta _{x_{kW}}\right| \le \eta ,\\ w_{k}\ge 0,\\ \sum w_{k}=1. \end{array} \right. \end{aligned},$$

where $k=1,2,\ldots ,n$. Next, we will give an example to show how this model works.

**Table 1:** The maximum value of $\xi$ about *x*.

$\alpha _{x_{BW}}$	1	2	3	4	5	6	7	8	9
$\xi$	0	0.4384	1.0000	1.6277	2.2984	3.0000	3.7251	4.4689	5.2280

**Table 2:** The maximum value of $\xi$ about *y*.

$\beta _{x_{BW}}$	1	1/2	1/3	1/4	1/5	1/6	1/7	1/8	1/9
$\eta$	0	2.1180	1.7908	1.6160	1.5062	1.4304	1.3747	1.3321	1.2519

Here, we denote max $\xi$ by $\alpha$ consistency index ($\alpha$-CI) $\xi ^{*}$ and max $\eta$ by $\beta$ consistency index ($\beta$-CI) $\eta ^{*}$. In addition, the consistent ratios value are $CR_{\alpha }=\frac{\xi ^{*}}{CI}$, where the value of CI depends on the value of $\alpha _{x_{BW}}$, detailed values shown in Table 1 and $CR_{\beta }=\frac{\eta ^{*}}{CI}$, where the value of CI depends on the value of $\beta _{x_{BW}}$, detailed values shown in Table 2.

### A TOPSIS method with a novel distance measure

For any two IFNs $\alpha (\mu _{\alpha },\nu _{\alpha },\pi _{\alpha })$ and $\beta (\mu _{\beta },\nu _{\beta },\pi _{\beta })$, in order to calculate their difference degree, we would propose a novel distance measure ${\overline{h}}(\alpha ,\beta )$. Chen and Tan^54^ proposed a score function $S(b_{ij}^{t})=\mu _{b_{ij}^{t}}-\nu _{b_{ij}^{t}}$ to calculate the score value of an intuitionistic fuzzy number $a_{b_{ij}^{t}}=(\mu _{b_{ij}^{t}},\nu _{b_{ij}^{t}})$, where $-1\le S(b_{ij}^{t})\le 1$. Hong et al.^55^ noted out that the scoring function alone cannot achieve the purpose of comparing different intuitionistic fuzzy numbers; that is, the score function will fail. For example, $a_{1}=(0.75,0.15)$ and $a_{2}=(0.68,0.08)$, and their score values are the same; $S(a_{1})=S(a_{2})=0.60$. However, it is clear that the evaluation results of these two evaluations are different. Hong et al.^55^ defined the accuracy function of intuitionistic fuzzy numbers to differentiate them. For an IFN $a=(\mu _{b},\nu _{b})$, $H(a)=\mu _{b}+\nu _{b}$ is its accuracy function. Then, we can obtain the hesitation degree by $\pi (a) =1-(\mu +\nu )=1-H(a)$. For the above example, we can calculate the accuracy values $H(a_{1})=0.90$ and $H(a_{2})=0.76$, obtaining $a_{1}>a_{2}$. This means $a_{1}$ is better. In summary, Xu and Yager^56^ gave a comparison method for any two different intuitionistic fuzzy numbers $a_{1}$ and $a_{2}$.

If $S(a_{1})<S(a_{2})$, then $a_{1}<a_{2}$, which means that $a_{1}$ is smallar than $a_{2}$;

If $S(a_{1})>S(a_{2})$, then $a_{1}>a_{2}$, which means that $a_{1}$ is larger than $a_{2}$;

If $S(a_{1})=S(a_{2})$: and if $H(a_{1})<H(a_{2})$, then $a_{1}<a_{2}$, which means that $a_{1}$ is smaller than $a_{2}$;

If $H(a_{1})>H(a_{2})$, then $a_{1}>a_{2}$, which means that $a_{1}$ is larger than $a_{2}$; and if $H(a_{1})=H(a_{2})$, then $a_{1}=a_{2}$, which means that $a_{1}$ is equal to $a_{2}$.

The distance difference degree ${\overline{h}}(\alpha ,\beta )$ can be calculated by:

19 $$\begin{aligned} {\overline{h}}(\alpha , \beta ) =|\mu _{A}(x_{i})-\mu _{B}(x_{i})| +|\nu _{A}(x_{i})-\nu _{B}(x_{i})| +|\pi _{A}(x_{i})-\pi _{B}(x_{i})| +|S_{A}(x_{i})-S_{B}(x_{i})| \end{aligned}.$$

Before proposing the novel method, we firstly introduce some basic concepts and methods which will be needed to construct the new model. Note that $X=\{x_{1},x_{2},\ldots ,x_{n}\}$ is a set of *n* alternatives; $C=\{c_{1},c_{2},\ldots ,c_{m}\}$ is a set of *m* criteria; $W=\{w_{1},w_{2},\ldots ,w_{m}\}$ is a set of weights corresponding to the set *C*; $E=\{e_{1},e_{2},\ldots ,e_{T}\}$ is a set of decision makers; $D=\{\lambda _{1},\lambda _{2},\ldots ,\lambda _{T}\}$ is a set of decision makers’ weights. In addition, the distance measure $h(\alpha ,\beta )$ yields the following propositions about $d_{IMN}$.

Let *A*, *B* and *C* be any three intuitionistic fuzzy sets. $\alpha$, $\beta$ and $\gamma$ are three IFNs from *A*, *B* and *C*, respectively. ${\overline{h}}(\alpha ,\beta )$ is the proposed distance measure, which has the following properties:

1. $${\overline{h}}(\alpha ,\beta )$$ is boundary;
2. $${\overline{h}}(\alpha ,\beta )=0$$ iff $$\alpha =\beta$$;
3. $${\overline{h}}(\alpha ,\beta )={\overline{h}}(\beta ,\alpha )$$.

#### Proof

1. ${\overline{h}}(\alpha ,\beta ) =|\mu _{A}(\alpha )-\mu _{B}(\beta )|+|\nu _{A}(\alpha )-\nu _{B}(\beta )| +|\pi _{A}(\alpha )-\pi _{B}(\beta )|+|S_{A}(\alpha )-S_{B}(\beta )|.$ Considering the boundaries of intuitionistic fuzzy numbers and their elements, get $0\le \mu _{A}(\alpha ),\nu _{A}(\alpha ),\pi _{A}(\alpha )\le 1$, $S_{A}(\alpha )=\mu _{A}(\alpha )-\nu _{A}(\alpha )$, $S_{B}(\alpha )=\mu _{B}(\alpha )-\nu _{B}(\alpha )$, then $-1\le S_{A}(\alpha )\le 1$, $-1\le S_{B}(\beta )\le 1$, $0\le |S_{A}(\alpha )-S_{B}(\beta )|\le 2$; along with $0\le |\mu _{A}(\alpha )-\mu _{B}(\beta )|\le 1$, $0\le |\nu _{A}(\alpha )-\nu _{B}(\beta )|\le 1$, $0\le |\pi _{A}(\alpha )-\pi _{B}(\beta )|\le 1$, obtain $0\le {\overline{h}}(\alpha ,\beta )\le 5$.
  Especially, let $\widetilde{{h}}(\alpha ,\beta ) =\frac{1}{4}(|\mu _{A}(\alpha )-\mu _{B}(\beta )|+|\nu _{A}(\alpha )-\nu _{B}(\beta )| +|\pi _{A}(\alpha )-\pi _{B}(\beta )|+\frac{1}{2}|S_{A}(\alpha )-S_{B}(\beta )|)$ then $0\le |\widetilde{{h}}(\alpha ,\beta )|\le 1$.
2. If ${\overline{h}}(\alpha ,\beta )=0$, then consider the definition of ${\overline{h}}(\alpha ,\beta )$ and
  20 $$\begin{aligned} \left\{ \begin{aligned}&|\mu _{A}(\alpha )-\mu _{B}(\beta )|\ge 0,\\&|\nu _{A}(\alpha )-\nu _{B}(\beta )|\ge 0,\\&|\pi _{A}(\alpha )-\pi _{B}(\beta )|\ge 0,\\&|S_{A}(\alpha )-S_{B}(\beta )|\ge 0,\\ \end{aligned} \right. \end{aligned}$$
  and get
  21 $$\begin{aligned} \left\{ \begin{aligned}&|\mu _{A}(\alpha )-\mu _{B}(\beta )|=0,\\&|\nu _{A}(\alpha )-\nu _{B}(\beta )|=0,\\&|\pi _{A}(\alpha )-\pi _{B}(\beta )|=0,\\&|S_{A}(\alpha )-S_{B}(\beta )|=0,\\ \end{aligned} \right. \end{aligned}$$
  that means $$\mu _{A}(\alpha )=\mu _{B}(\beta )$$, $$\nu _{A}(\alpha )=\nu _{B}(\beta )$$, $$\pi _{A}(\alpha )=\pi _{B}(\beta )$$, i.e., $$\alpha =\beta$$.
3. ${\overline{h}}(\alpha ,\beta )={\overline{h}}(\beta ,\alpha )$, that is obvious.

Equations (5)–(7) is proposed by reference^57^, which may exist powerless conditions. Such as shown in Example 1, these functions cannot class $P_{1}$ and $P_{2}$. Equation (16) is an extension of Eq. (7), and not able to deal with Example 2. The following Table 3 could show this paper’s class results are the same with different methods. $\square$

#### Example 1

There are two patterns $P_{1}$ and $P_{2}$ represented by IFNs $\widetilde{P_{1}}$ and $\widetilde{P_{2}}$ in the universe of $X=\{x_{1},x_{2},x_{3}\}$,

$$\widetilde{P_{1}}=\{\langle x_{1},0.6,0.25\rangle ,\langle x_{2},0,0.25\rangle ,\langle x_{3},0.3,0.25\rangle \}$$;

$$\widetilde{P_{2}}=\{\langle x_{1},0.1,0.75\rangle ,\langle x_{2},0.15,0.1\rangle ,\langle x_{3},0.2,0.35\rangle \}$$.

Here is an unknown pattern represented by an IFS ${\widetilde{Q}}$ to one of $\widetilde{P_{1}}$ and $\widetilde{P_{2}}$, where

$${\widetilde{Q}}=\{\langle x_{1},0,0.15\rangle ,\langle x_{2},0.325,0.425\rangle ,\langle x_{3},0.2,0.25\rangle \}$$.

**Table 3:** Comparison results for different distance measures.

*Method*	$D_{1}(P_{1},Q)$	$D_{2}(P_{2},Q)$	Comparison	Classing result
Reference^58^	0.1278	0.1278	$D_{1}(P_{1},Q)=D_{2}(P_{2},Q)$	Q could not be determined
Reference^59^	0.2796	0.2354	$D_{1}(P_{1},Q)\ge D_{2}(P_{2},Q)$	Q should be classified into team $P_{2}$
This paper’s	1.3750	1.3000	$D_{1}(P_{1},Q)\ge D_{2}(P_{2},Q)$	Q should be classified into team $P_{2}$

For each criterion $c_{l}$, $l\in \{1,2,\ldots ,m\}$, DM $e_{t}$, $t\in \{1,2,\ldots ,T\}$ chooses the best alternative $x_{B}$ and the worst $x_{W}$. And give the comparison results: $X^{l}_{B(t)}=(x^{l}_{B1},x^{l}_{B2},\ldots ,x^{l}_{Bn})$, $X^{l}_{W(t)}=(x^{l}_{1W},x^{l}_{2W},\ldots ,x^{l}_{nW})$, where $x^{l}_{Bj}$ and $x^{l}_{iW}$, $i,j\in \{1,2,\ldots ,n\}$ are IMNs, which stand for the preferred degree of $x_{B}$ over $x_{j}$ and $x_{i}$ over $x_{W}$ with respect to the criterion $c_{l}$. Then, obtain the decision matrixes $DM_{B(t)}=(X^{l}_{B(t)})_{n\times m}$ and $DM_{W(t)}=(X^{l}_{W(t)})_{n\times m}$. Next are the steps of the proposed TOPSIS based on the Eq. (19).

Step 1: Under criterion $C_{j}$, determine the positive solution $b_{j}^{+}=(\mu _{b_{j}^{+}},\nu _{b_{j}^{+}})$ and negative solution $b_{j}^{-}=(\mu _{b_{j}^{-}},\nu _{b_{j}^{-}})$ :
  22 $$b_{j}^{ + } = \left\{ {\begin{array}{*{20}l} {(max\mu _{{b_{{ij}} }} ,min\nu _{{b_{{ij}} }} ),} \hfill & {j \in J_{1} ,} \hfill \\ {(min\mu _{{b_{{ij}} }} ,max\nu _{{b_{{ij}} }} ),} \hfill & {j \in J_{2} .} \hfill \\ \end{array} } \right.$$
  23 $$b_{j}^{ - } = \left\{ {\begin{array}{*{20}l} {(min\mu _{{b_{{ij}} }} ,max\nu _{{b_{{ij}} }} ),} \hfill & {j \in J_{1} ,} \hfill \\ {(max\mu _{{b_{{ij}} }} ,min\nu _{{b_{{ij}} }} ),} \hfill & {j \in J_{2} .} \hfill \\ \end{array} } \right.$$
  where, $J_{1}$ represents an efficiency evaluation index set, $J_{2}$ represents the cost evaluation index set.
Step 2: Calculate the distance between each of the alternatives in the public project and the positive ideal and the negative ideal. This calculation is performed using a weighted Euclidean distance formula that considers the score function. Depending on the proposed distance measure proposed in Eq. (19), we construct the Model 2 for the positive and negative distance measures.

**Model 2**

Calculate the distance from each solution to the positive ideal solution:

24 $$\begin{aligned} d_{i}^{+}=\bigg [\sum _{j=1}^{n}w_{j}\bigg ( \bigg |\mu _{i}(x_{j})-\mu _{b_{j}^{+}}\bigg | +\bigg |\nu _{i}(x_{j})-\nu _{b_{j}^{+}}\bigg | +\bigg |\pi _{i}(x_{j})-\pi _{d_{j}^{+}}\bigg | +\bigg |S_{i}(x_{j})-S_{d_{j}^{+}}\bigg | \bigg )^{2}\bigg ]^{\frac{1}{2}} \end{aligned}.$$

Calculate the distance from each solution to the negative ideal solution:

25 $$\begin{aligned} d_{i}^{-}=\bigg [\sum _{j=1}^{n}w_{j}\bigg ( \bigg |\mu _{i}(x_{j})-\mu _{b_{j}^{-}}\bigg | +\bigg |\nu _{i}(x_{j})-\nu _{b_{j}^{-}}\bigg | +\bigg |\pi _{i}(x_{j})-\pi _{d_{j}^{-}}\bigg | +\bigg |S_{i}(x_{j})-S_{d_{j}^{-}}\bigg | \bigg )^{2}\bigg ]^{\frac{1}{2}} \end{aligned}.$$

Step 3: Calculate the integrated distance:
  26 $$\begin{aligned} R_{i}=\frac{d_{i}^{-}}{d_{i}^{+}+d_{i}^{-}} \end{aligned},$$
  where $i=1,2,\cdots ,m$ is the number of alternatives.
Step 4: Rank the alternatives according to their rations.

Depending on the meanings of the distances $d_{j}^{+}$ and $d_{j}^{-}$, the larger the value of $R_{i}$ is, the better the alternative $X_{i}$; the smaller the value of $R_{i}$ is, the worse the alternative $X_{i}$. Therefore, we can sort public project programs and select the best program according to the number of integrated distances selected.

#### Example 2

**Figure 3:**
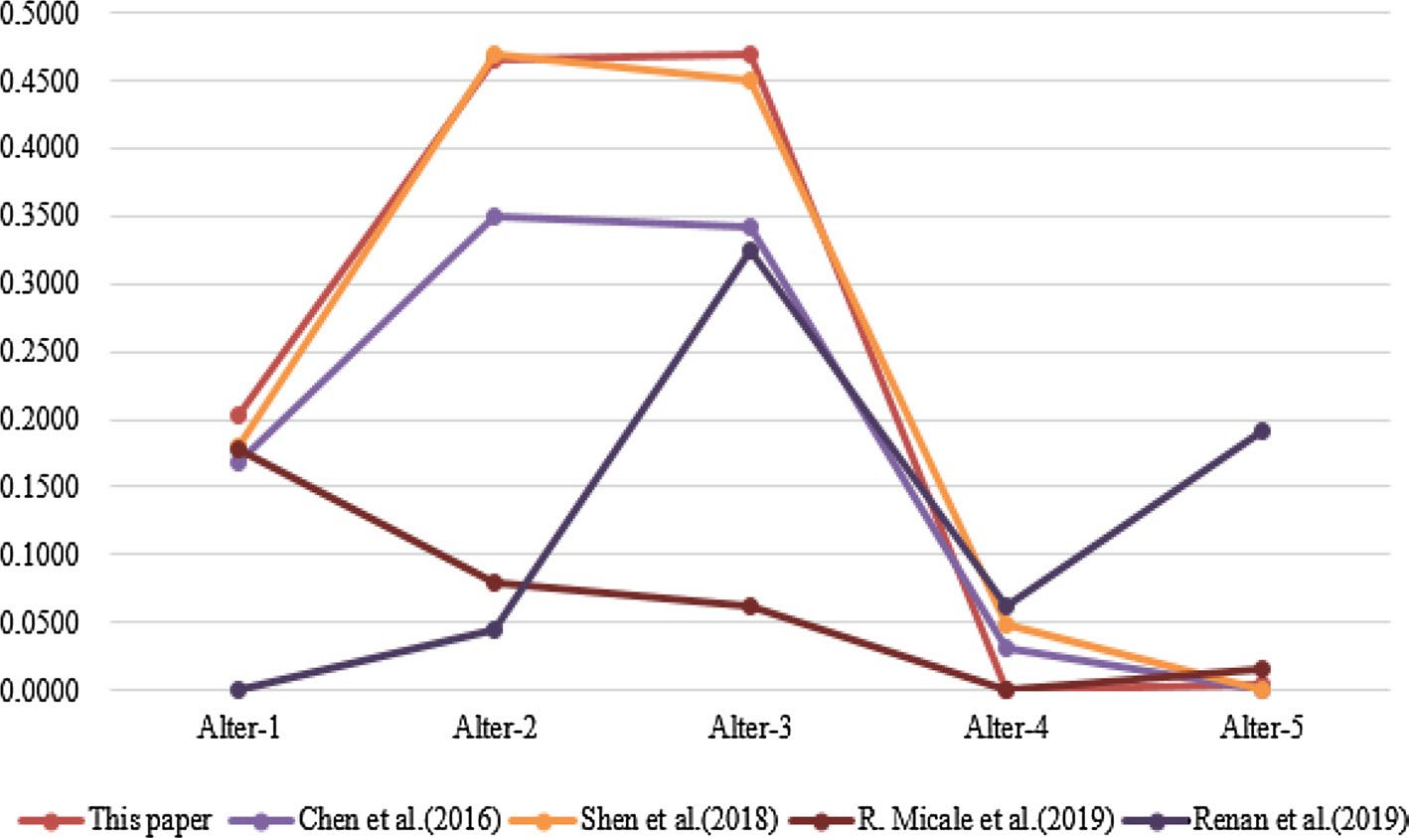
Comparison results of different TOPSIS methods.

**Figure 4:**
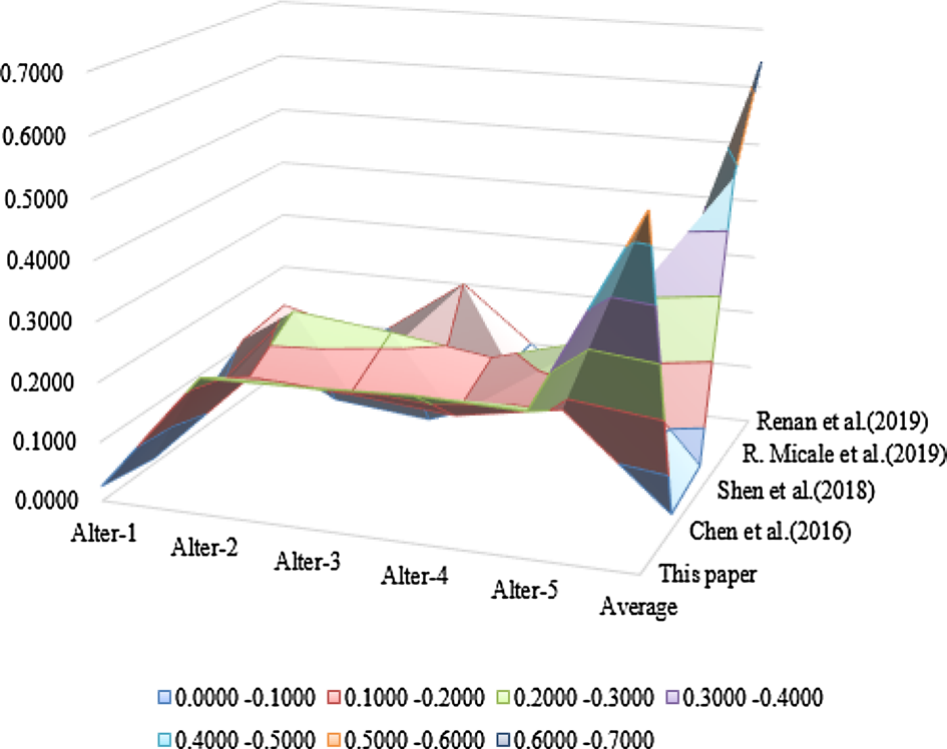
Change range of different Topsis methods.

An illustrative example from preference^60^, shown in Table 4, is given to indicate the effectiveness of the proposed method. This example was also used in preference^39^ and preference^61^ and preference^62^. Their results are compared in Figs. 3 and 4. From Fig. 3, we could see that the proposed TOPSIS method’s ranking results are similar with the others. Figure 4 shows the error distribution comparisons between the different methods, where the higher the deviation value is, the better the separation ability, and the results indicate that the proposed method is more effective.

**Table 4:** A example to show the different distance measures based on IFNs.

	Criterion 1	Criterion 2	Criterion 3	Criterion 4	Criterion 5
Alter-1	(0.6, 0.3)	(0.4, 0.3)	(0.3, 0.5)	(0.6, 0.3)	(0.7, 0.2)
Alter-2	(0.6, 0.2)	(0.5, 0.3)	(0.5, 0.1)	(0.6, 0.3)	(0.6, 0.1)
Alter-3	(0.8, 0.1)	(0.3, 0.2)	(0.6, 0.1)	(0.5, 0.4)	(0.5, 0.1)
Alter-4	(0.7, 0.2)	(0.1, 0.1)	(0.5, 0.3)	(0.2, 0.5)	(0.5, 0.6)
Alter-5	(0.6, 0.2)	(0.5, 0.6)	(0.3, 0.4)	(0.2, 0.5)	(0.6, 0.2)

### Two-sided method based on BWM and TOPSIS

#### Two-sided matching

The mathematical definition of matching is derived from one-to-one mapping^63^. Let $A=\{A_{1},A_{2},\ldots ,A_{m}\}$ and $B=\{B_{1},B_{2},\ldots ,B_{n}\}$ be two sets, with *m* and *n* elements, separately.

**Figure 5:**
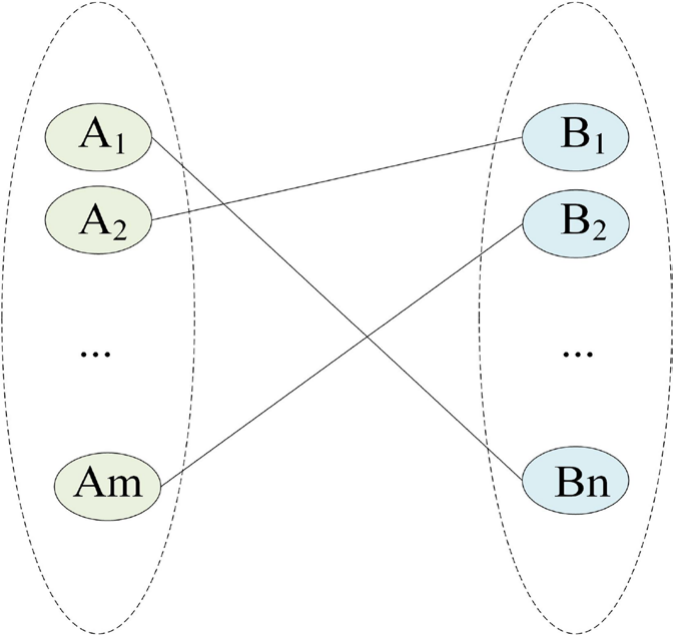
Two-sided matching.

One-to-one mapping $\theta$: $A\cup B\rightarrow A\cup B$ be a two-sided matching iff $\forall A_{i}\in A$, $i\in M$, $\forall B_{j}\in B$, $j\in N$ satisfy the following conditions, shown in Fig. 5:

1. $$\theta (A_{i})\in B$$, $$i\in M$$;
2. $$\theta (B_{j})\in A\cup \{B_{j}\}$$, $$j\in N$$;
3. $$\theta (A_{i})=B_{j}$$ iff $$\theta (B_{j})=A_{i}$$, $$i\in M$$, $$j\in N$$.

If $\theta (A_{i})=B_{j}$, then $(A_{i},B_{j})$ is called a matching couple under one-to-one mapping $\theta$; If $\theta (A_{i})\ne B_{j}$, then $(A_{i}$ is called un-matching under one-to-one mapping $\theta$. Let one-to-one mapping $\theta$: $A\cup B\rightarrow A\cup B$ be a two-sided matching iff $\forall A_{i}\in A$, $i\in M$, $\forall B_{j}\in B$, $j\in N$ satisfy the following conditions:

1. $$\theta (A_{i})\in B$$, $$i\in M$$;
2. $$\theta (B_{j})\in A\cup \{B_{j}\}$$, $$j\in N$$;
3. $$\theta (A_{i})=B_{j}$$ iff $$\theta (B_{j})=A_{i}$$, $$i\in M$$, $$j\in N$$.

**Figure 6:**
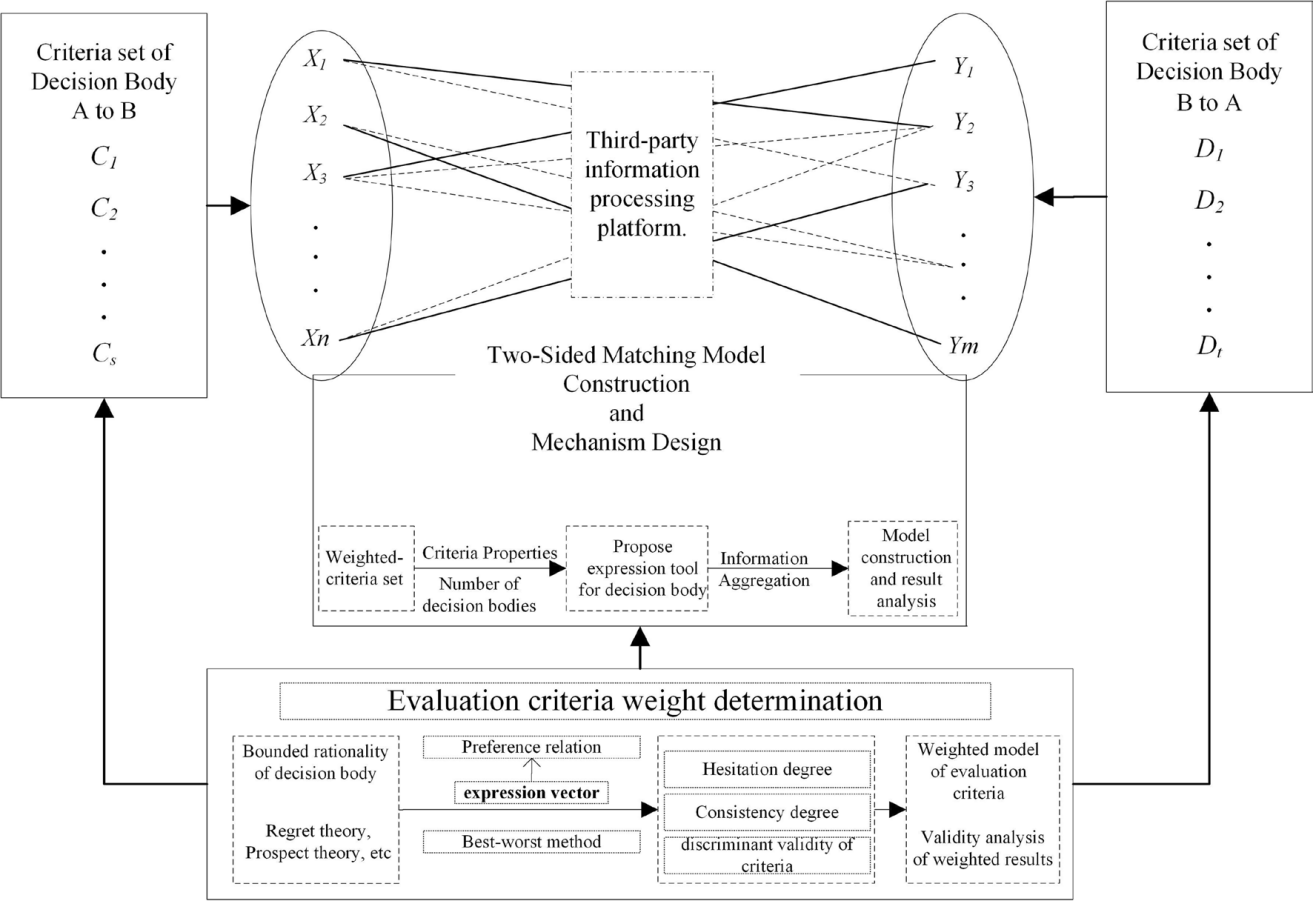
Two-sided matching process.

If all the elements in set A correspond to elements in set B through this mapping and the elements in set B can also be used to map all the elements in set A, then set A and set B are a two-sided match, as shown in Fig. 6.

#### Two-sided method based on BWM and TOPSIS

Let $A=\{A_{1},A_{2},\ldots ,A_{m}\}$ be a set with *m* decision makers and $B=\{B_{1},B_{2},\ldots ,B_{n}\}$ be a set with *n* decision makers.

$C=\{C_{1},C_{2},\ldots ,C_{F}\}$ is the criteria set for decision maker $A_{i}$ to evaluate $B_{j}$. $D=\{D_{1},D_{2},\ldots ,D_{G}\}$ is the criteria set for decision maker $B_{j}$ to evaluate $A_{i}$. Each member $A_{i}$ from set *A* describes preference degree to member $B_{j}$ from set *B* under criterion $C_{f}$ by the I-BWM, obtaining $s_{Bj}=(\rho _{Bj},\sigma _{Bj})$ and $s_{iW}=(\rho _{iW},\sigma _{iW})$. Each member $B_{j}$ from set *A* describe preference degree to member $A_{i}$ from set *B* under criterion $C_{g}$, obtaining $t_{Bj}=(\rho _{Bj},\sigma _{Bj})$ and $t_{iW}=(\rho _{iW},\sigma _{iW})$.

Taking all criteria and members into consideration, we obtain the satisfaction degree matrix $A(B)_{Best}=(s_{Bj}^{f})_{n\times f}$, $A(B)_{Worst}=(s_{iW}^{f})_{n\times f}$ and $B(A)_{Best}=(t_{Bj}^{g})_{m\times g}$, $B(A)_{Worst}=(t_{iW}^{g})_{m\times g}$.

Calculating the consistence degree of decision vectors based on Model 1, we can obtain the weight vector $W(A_{m})_{Best}=(C_{1},C_{2},\ldots ,C_{F})_{Best}$ and the weight vector $W(A_{m})_{Worst}=(C_{1},C_{2},\ldots ,C_{F})_{Worst}$ of alternatives from set *B* under criterion $C_{f}$ by decision maker $A_{i}$; also can obtain the weight vector $W(B_{n})_{Best}=(D_{1},D_{2},\ldots ,D_{G})_{Best}$ and the weight vector $W(B_{n})_{Worst}=(D_{1},D_{2},\ldots ,D_{F})_{Worst}$ of alternatives from set *A* under criterion $D_{g}$ by decision maker $B_{i}$.

Apply Eq. (15), we obtain the decision matrix $\overline{A(B)}_{Best}=((r_{ij}^{f})_{Best})_{n\times f}$, $\overline{A(B)}_{Worst}=((r_{ij}^{f})_{Worst})_{n\times f}$ Considering all of the criteria, we could obtain satisfactory-degree vector $S^{j}(A_{i})_{Best}=((d_{ij})_{Best})_{n\times f}$ for alternative $A_{i}$ and satisfactory-degree vector $S^{j}(A_{i})_{Worst}=((d_{ij})_{Worst})_{n\times f}$ for alternative $A_{i}$; satisfactory-degree vector $S^{j}(B_{j})_{Best}=((e_{ij})_{Best})_{n\times f}$ for alternative $B_{j}$ and satisfactory-degree vector $S^{j}(B_{j})_{Worst}=((e_{ij})_{Worst})_{n\times f}$ for alternative $B_{j}$.

Then we can get the satisfactory decision matrix $A(B)=((d_{ij})_{Best})n\times m$ of decision body *A*, where $(d_{ij})_{Best}=\sum _{f}(w(C_{f})r_{ij}^{f})_{Best}$; the satisfactory decision matrix $A(B)=((d_{ij})_{Worst})n\times m$ of decision body *A*, where $(d_{ij})_{Worst}=\sum _{f}(w(C_{f})r_{ij}^{f})_{Worst}$.

And the satisfactory decision matrix $B(A)=((e_{ij})_{Best})_{m\times n}$ of decision body *B*, where $(e_{ij})_{Best}=\sum _{g}(w(D_{g})r_{ij}^{g})_{Best}$; the satisfactory decision matrix $B(A)=((e_{ij})_{Worst})_{m\times n}$ of decision body *B*, where $(e_{ij})_{Worst}=\sum _{g}(w(D_{g})r_{ij}^{g})_{Worst}$.

**Model 3**:

27 $$\begin{aligned} max Z(A) =\sum _{i=1}^{m}\sum _{j=1}^{n}w(A_{i})_{Best}\sum _{j=1}^{n}(d_{ij})_{n\times m} \end{aligned}.$$

s.t.

28 $$\begin{aligned}&max Z(B) =\sum _{i=1}^{m}\sum _{j=1}^{n}w(B_{j})_{Best}\sum _{i=1}^{m}(e_{ij})_{n\times m} \end{aligned},$$

29 $$\begin{aligned}&\sum _{i=1}^{m}x_{ij}=1 \end{aligned},$$

30 $$\begin{aligned}&\sum _{j=1}^{n}x_{ij}\le 1 \end{aligned},$$

31 $$\begin{aligned}&x_{ij}\in \{0,1\}, i\in M, j\in N, \end{aligned}$$

where $x_{ij}=0$ means $A_{i}$ and $B_{j}$ does not match, and $x_{ij}=1$ means $A_{i}$ and $B_{j}$ match.

**Model 4**

32 $$\begin{aligned} max Z(A) =\sum _{i=1}^{m}\sum _{j=1}^{n}w(A_{i})_{Worst}\sum _{j=1}^{n}(d_{ij})_{n\times m} \end{aligned},$$

s.t.

33 $$\begin{aligned}&max Z(B) =\sum _{i=1}^{m}\sum _{j=1}^{n}w(B_{j})_{Worst}\sum _{i=1}^{m}(e_{ij})_{n\times m} \end{aligned},$$

34 $$\begin{aligned}&\sum _{i=1}^{m}x_{ij}=1 \end{aligned},$$

35 $$\begin{aligned}&\sum _{j=1}^{n}x_{ij}\le 1 \end{aligned},$$

36 $$\begin{aligned}&x_{ij}\in \{0,1\}, i\in M, j\in N \end{aligned},$$

where $x_{ij}=0$ means $A_{i}$ and $B_{j}$ does not match, and $x_{ij}=1$ means $A_{i}$ and $B_{j}$ match.

In order to solve the Model 3 and Model 4, we introduce the following Model 5 and Model 6.

**Model 5**

37 $$\begin{aligned} max Z=\alpha Z(A)_{Best}+\beta Z(B)_{Best} \end{aligned},$$

s.t.

38 $$\begin{aligned}&\sum _{i=1}^{m}x_{ij}=1 \end{aligned},$$

39 $$\begin{aligned}&\sum _{j=1}^{n}x_{ij}\le 1 \end{aligned},$$

40 $$\begin{aligned}&x_{ij}\in \{0,1\}, i\in M, j\in N \end{aligned}.$$

**Model 6**

41 $$\begin{aligned} max Z=\alpha Z(A)_{Worst}+\beta Z(B)_{Worst} \end{aligned}.$$

s.t.

42 $$\begin{aligned}&\sum _{i=1}^{m}x_{ij}=1 \end{aligned},$$

43 $$\begin{aligned}&\sum _{j=1}^{n}x_{ij}\le 1 \end{aligned},$$

44 $$\begin{aligned}&x_{ij}\in \{0,1\}, i\in M, j\in N \end{aligned}.$$

Taking the evaluations based on the Best-alternative and Worst-alternatives into consideration at the same time, we introduce the Model 7.

**Model 7**

45 $$\begin{aligned} max Z=\lambda Z_{Best}+(1-\lambda )Z_{Worst} \end{aligned}.$$

s.t.

46 $$\begin{aligned}&\sum _{i=1}^{m}x_{ij}=1 \end{aligned},$$

47 $$\begin{aligned}&\sum _{j=1}^{n}x_{ij}\le 1 \end{aligned},$$

48 $$\begin{aligned}&x_{ij}\in \{0,1\}, i\in M, j\in N \end{aligned},$$

where $\lambda$ is a coefficient that is determined by the deviation degree caused by the matching process based on the best alternative and the deviation degree caused by the matching process based on the worst alternative.

### Algorithm of the proposed two-sided decision method

**Figure 7:**
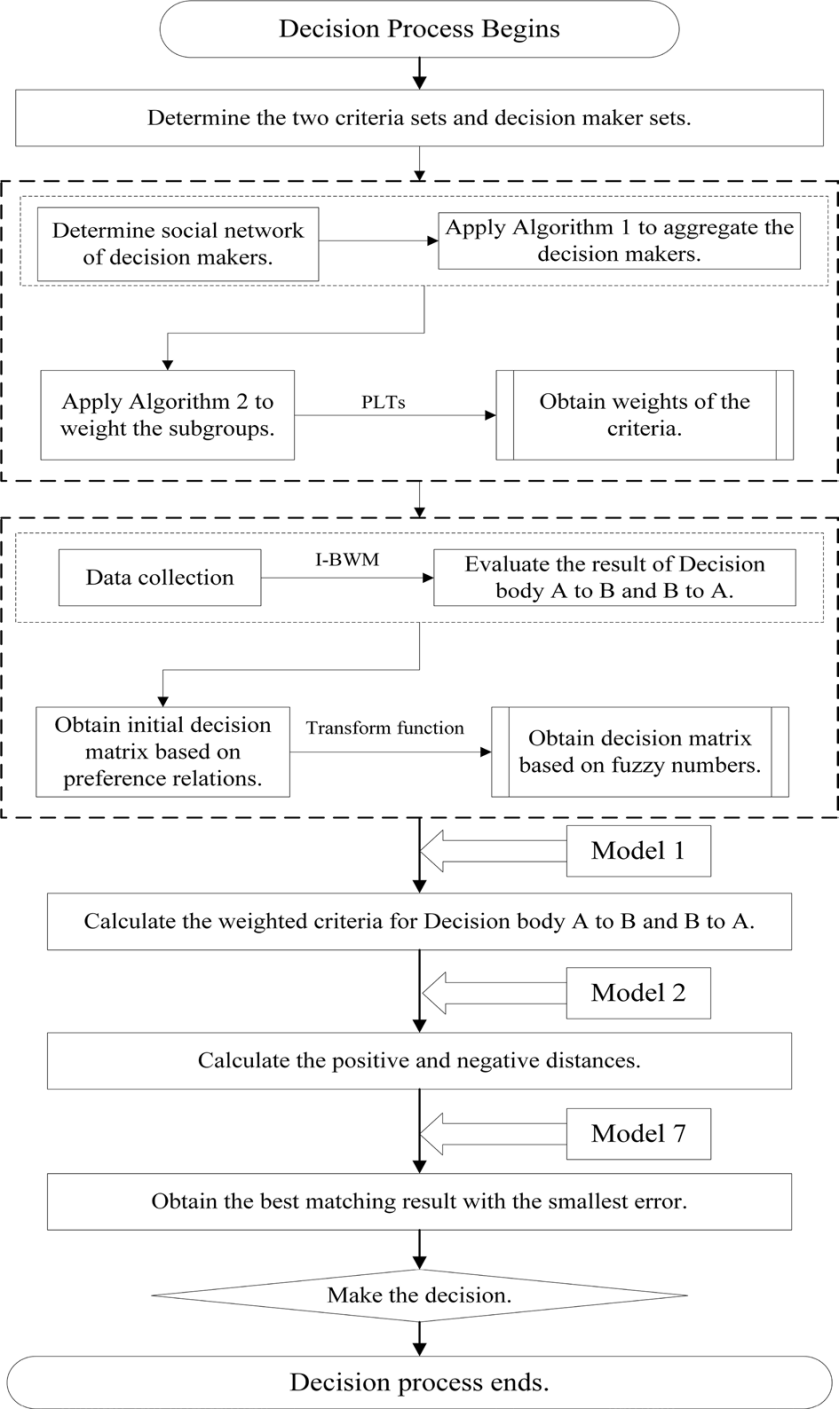
The proposed method’s decision making process.

On the basis of the models constructed in “Literature review” section, we summarize a novel two-sided matching process that is shown in Fig. 7. The decision process starts from the determination of the alternatives set *X* and criteria set *C*. Decision bodies *A* and *B* will evaluate each other considering set *C*, with respect to the alternatives. The initial decision results are obtained by the $I-BWM$. We propose a transformation function to obtain the decision matrix based on fuzzy numbers. Then, the evaluating information will be aggregated more effectively. Next, Model 1 is introduced to weight the criteria, Model 2 is introduced to calculate the positive and negative distances, and Model 7 is introduced to obtain the best matching result with the smallest error. The decision process ends by obtaining the final matching result.

Step 1: The decision matrix is determined.
Step 2: Transform the evaluation result.
Step 3: Calculated the criteria weight vector.
Step 4: Apply the proposed TOPSIS method.
Step 5: Apply the proposed two-sided matching model.

## An illustrative example

To make the application more intuitive and concrete, the proposed model is applied to an international headhunting agency for matching high-level overseas talent and jobs in this section. First, building a scientific and reasonable evaluation index system is an important part of bilateral matching of people and jobs, and it is also the basis of the proposed model application. Therefore, on the basis of personnel evaluation theory and competency models, we adopted a literature research method and analyzed academic literature on science and technology talent^64^, high potential^65^, international talent^66^, high-level talent^67^, and so on. Then, we obtained the initial index of mutual evaluations between overseas talent and job positions, as shown in Table 5.

**Table 5:** The initial index of mutual evaluation between overseas talent and job position.

The evaluation index of job positions	The evaluation index of job seekers
Wage pay	Basic qualities
Incentives and promotion	Knowledge and Skill
Development potential	Performance
Job requirements	Business capabilities
Corporate culture	Development potential
Human resources management system	Internationalization
Organizational atmosphere	Teamwork and cooperation spirit

In particular, high-level talent mainly considers wage pay, incentives and promotion, postdevelopment potential, job requirements, corporate culture, the human resources management system, the organizational atmosphere and other aspects of a series of elements. The recruiter mainly analyzes the competency characteristics on the basis of the analysis of their past work, judges whether high-level overseas talent can be qualified for specific jobs and ensures excellent work performance in the positions.

Then, we used the Delphi method and collected questionnaire and interview information from 11 experts in the field of talent management. Through further consultations and adjustments, we obtained the consensus of the index set. job conditions are evaluated according to the evaluation index with 5 elements, consisting of salary and incentive, job development potential, job challenge, employer brand and corporate culture. The index of the recruiter in evaluating the high-level overseas talent consisted of the appropriateness of knowledge and skills, work experience and performance, internationalization and intercultural competence, consistency and difference of values, and expected performance. To be specific, the criteria are described here. Salary and incentive: The salary and reward for assuming the corresponding responsibility and the bonuses and promotions due to outstanding performance. Employer brand: The enterprise’s employer image, visibility and reputation, which are formed by providing quality and characteristic service for employees. Job challenge: The difficulties and pressure of completing the work scope, meeting the requirements, and taking job responsibility. Job development potential: The potential future earnings and personal development opportunities of the job position. Corporate culture: The unique mental outlook, the working environment atmosphere, and the humanistic atmosphere created in the daily operation of enterprises. Next, the criteria are explained specifically. Professional and technical fit degree: The knowledge and professional technology of high-level overseas talent and the consistency degree of the needed ability to complete the job tasks. Knowledge professional technology of work experience and performance: Related work experience, as well as contributions to the enterprise, industry, and even social development. Internationalization and intercultural competence: If high-level overseas talent has advanced international knowledge and skills, it has a global vision and ideas, can translate freely and has the ability to communicate in a multicultural environment. Consistency and difference of values: The consistency and difference of values, working manners and behavior. Expected performance: The expected tangible and intangible benefits of high-level overseas talent introduction.

### Criteria weights determination

There are 10 enterprise customers who want to introduce high-level overseas talent as senior executives, as technical supervisors and in the scenario of other positions. According to this headhunting agency searching in its international talent pool, there are 7 high-level overseas personnel have a preliminary intention.

**Figure 8:**
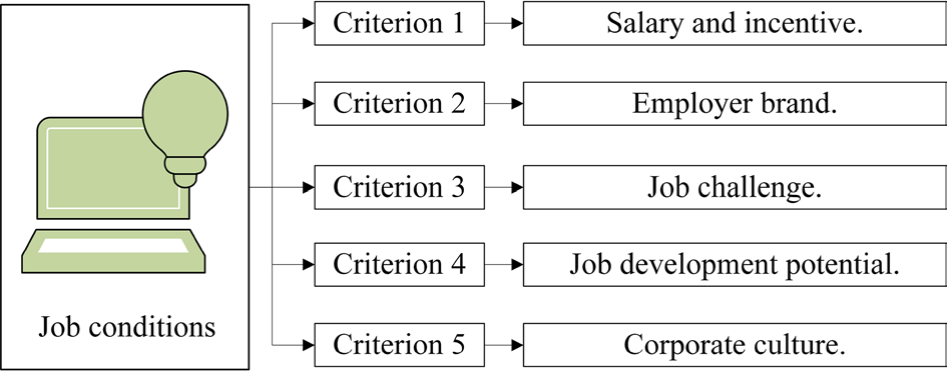
Criteria to evaluate job condition.

**Figure 9:**
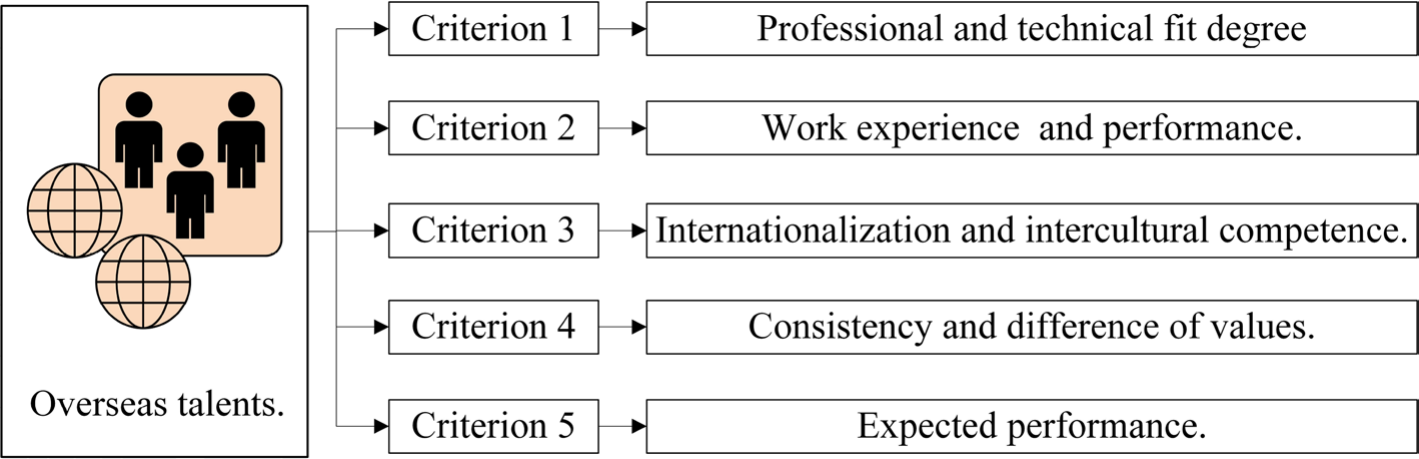
Criteria to evaluate overseas talent.

Utilize the LSGDM method to calculate decision makers’ aggregating results and criteria. We determine the alternatives, criteria set and decision makers. We have overseas talent and a decision body $A=\{A_{1},A_{2},\ldots ,A_{7}\}$ and $B=\{B_{1},B_{2},\ldots ,B_{10}\}$. The criteria that $A_{i}$ consider in evaluating decision body *B* are $C=\{C_{1},C_{2},\ldots ,C_{5}\}$, standing for 5 criteria as shown in Fig. 8. The criteria alternatives $B_{i}$ considered to evaluate decision body *A* are $D=\{D_{1},D_{2},\ldots ,D_{5}\}$, standing for 5 criteria, as shown in Fig. 9.

**Figure 10:**
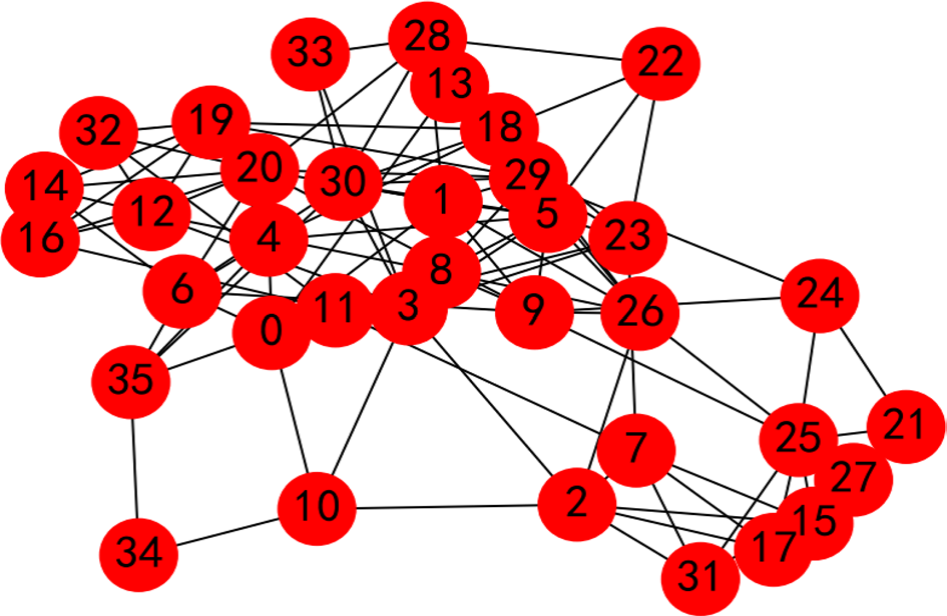
Decision makers’ social network for criteria to evaluate jobs.

**Figure 11:**
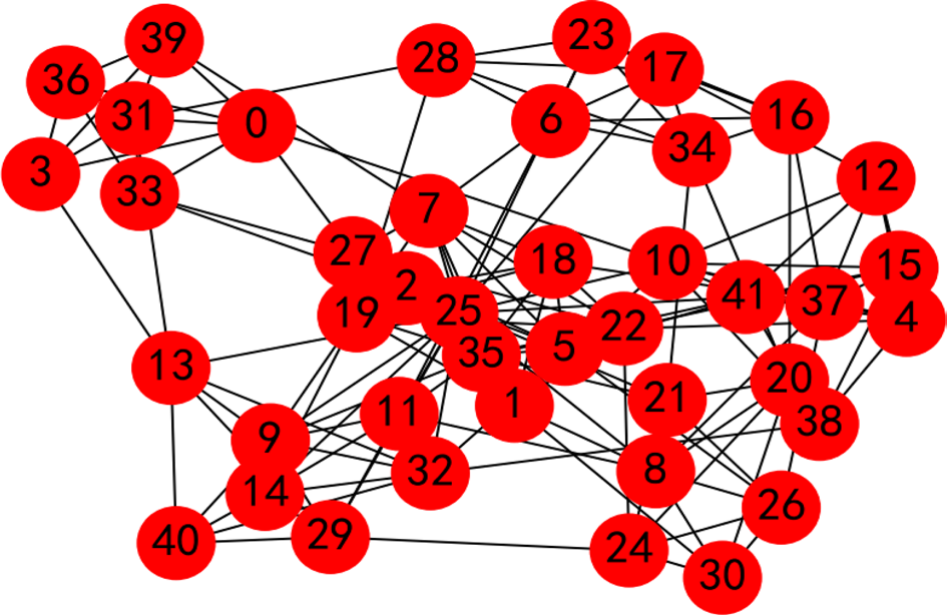
Decision makers’ social network for criteria to evaluate overseas talent.

We calculate the weight vector of the criteria with the proposed LSGDM method from “Literature review” section. Here, 35 decision makers are invited to evaluate the criteria for a job’s condition, whose social network is shown in Fig. 10; invite 41 decision makers are invited to evaluate the criteria for overseas talent, whose social network is shown in Fig. 11.

**Figure 12:**
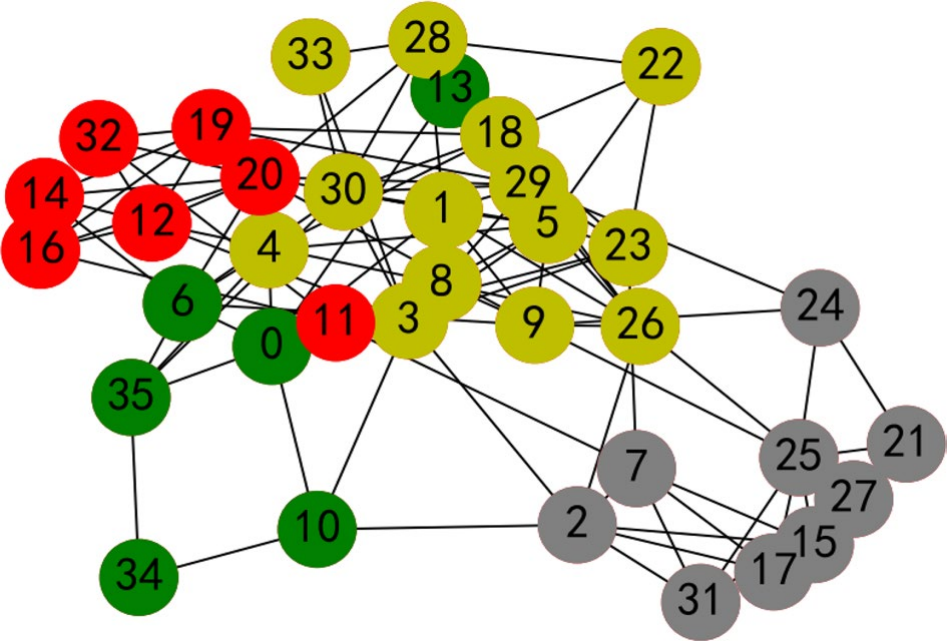
Aggregated results of the decision makers’ social network for criteria to evaluate jobs by the proposed method.

**Figure 13:**
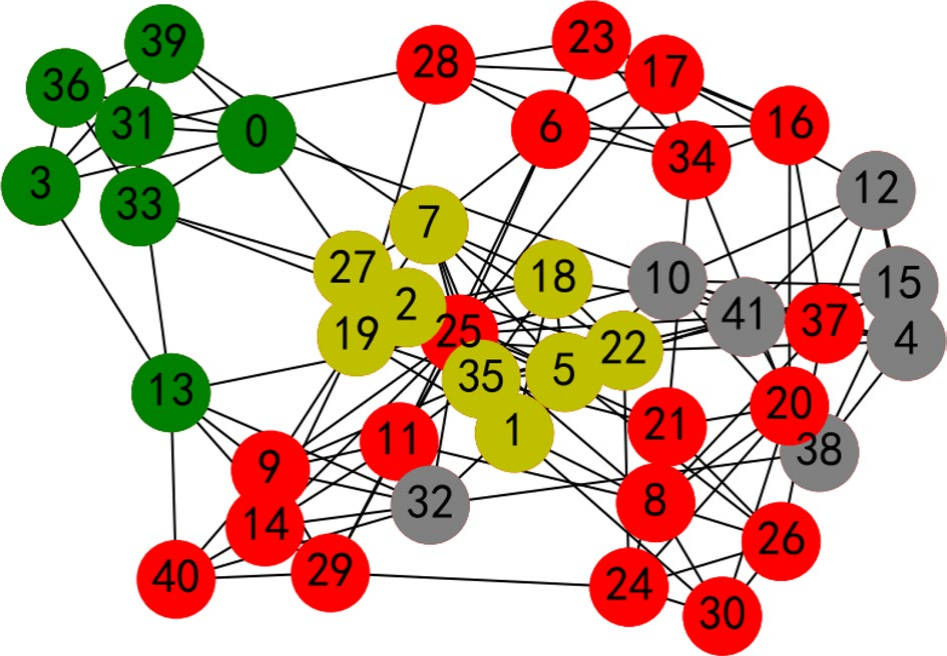
Aggregated results of the decision makers’ social network for criteria to evaluate overseas talent by the proposed method.

Applying Algorithm 1, the two decision making bodies are aggregated into some small subgroups, which are shown in Figs. 12 and 13.

Let each decision maker give the evaluation result in the form of PLTs, which are defined in Definition 5. In this example, let the linguistic terms be $\{s_{-3}.s_{-2},s_{-1},s_{0},s_{1},s_{2},s_{3}\}$, standing for the least important, less important, slightly less important, generally important, somewhat important, more important, and most important considerations . For example, Decision Maker 1 (DM1) is from the group that evaluates jobs, and his or her evaluation result is shown in Table 6 below.

**Table 6:** The decision results of criteria for evaluating overseas talents by $DM_{1}$.

	$C_{1}$	$C_{2}$	$C_{3}$	$C_{4}$	$C_{5}$
PLTs	$\{s_{-1}\left(\frac{1}{7}\right),s_{0}\left(\frac{6}{7}\right)\}$	$\{s_{0}\left(\frac{2}{7}\right),s_{1}\left(\frac{4}{7}\right)\}$	$\{s_{0}(1)\}$	$\{s_{2}\left(\frac{3}{7}\right),s_{3}\left(\frac{2}{7}\right)\}$	$\{s_{2}(1)\}$

**Figure 14:**
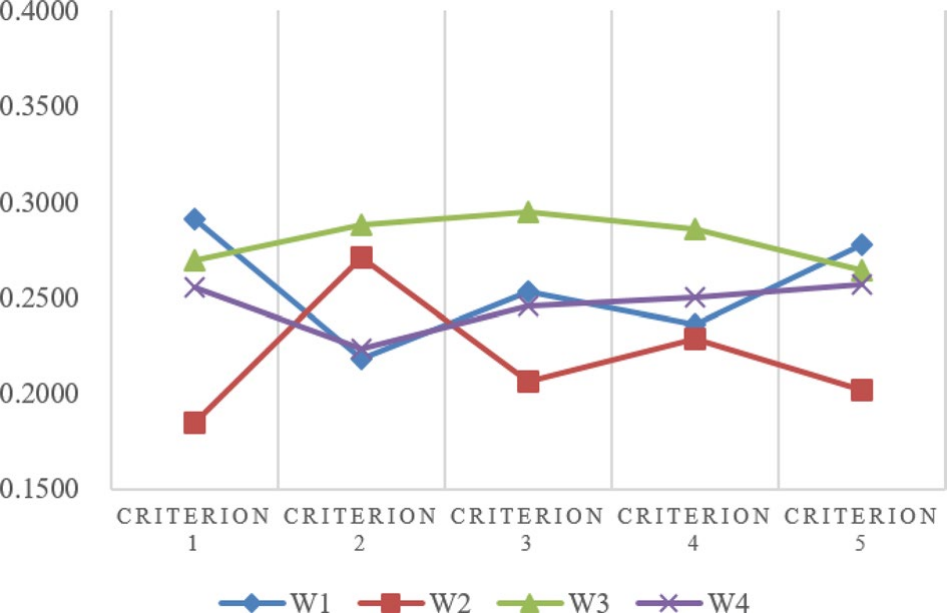
Criteria-weights of evaluating jobs.

**Figure 15:**
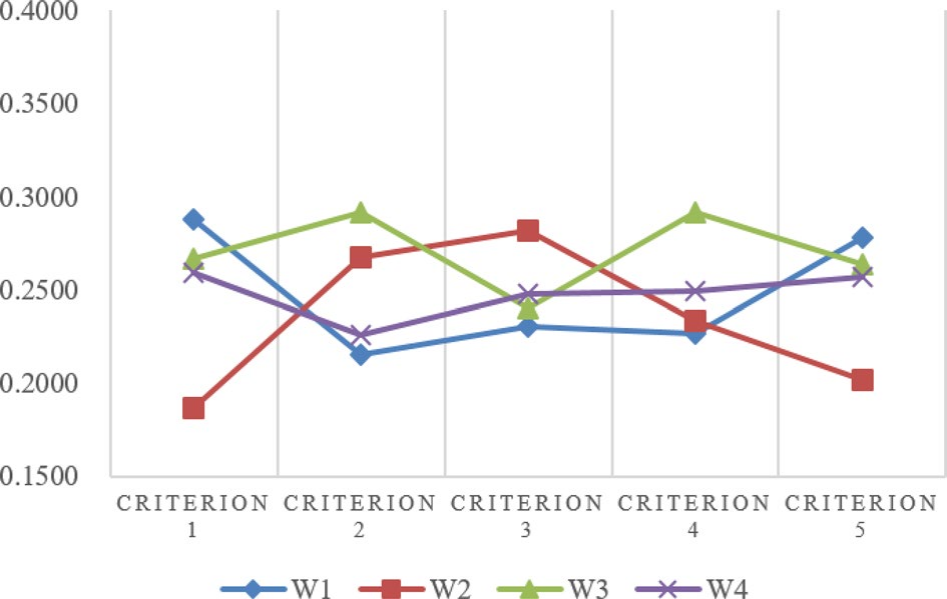
Criteria-weights of evaluating overseas talent.

By the definition of PLT and Theorem 1, the evaluation result’s hesitation degree is calculated. Each decision result’s expectation value can be calculated by the Eq. (3). A decision maker’s final integrated decision result is calculated by Eq. (4). Applying Algorithm 2, the subgroups’ weights are determined based on their positions. Then, the criteria weights can be obtained by the subgroups’ weights and their normalized final integrated decision results. The calculated weight vectors of the criteria with respect to job evaluation and overseas talent evaluation are shown in Figs.  14 and 15. Depending on the weights of the criteria, the procedure below is continued to obtain the final matching results.

### The proposed two sided decision making method

According to the I-BWM, $A_{i}$ evaluates alternatives $B_{1},B_{2},\ldots ,B_{10}$, choosing the best and worst alternatives and obtaining the evaluation results in the form of intuitionistic preference numbers; $B_{j}$ evaluates alternatives $A_{1}, A_{2},\ldots ,A_{10}$, choosing the best and worst alternatives and obtaining the evaluation results in the form of intuitionistic preference numbers. We transform the evaluation results to fuzzy preference relations under a multiplicative preference relation environment.

49 $$\begin{aligned} A_{1}(B)=\left[ \begin{array}{lllll} (9,1/9)(1,1) &{}(9,1/9)(1,1) &{}(8,1/9)(1,1) &{}(9,1/9)(1,1) &{}(9,1/9)(1,1) \\ (2,1/2)(8,1/8)&{}(3,1/3)(8,1/9)&{}(2,1/3)(7,1/8)&{}(3,1/4)(6,1/7)&{}(2,1/2)(7,1/8) \\ (1,1)(8,1/9) &{}(2,1/2)(8,1/8)&{}(3,1/4)(6,1/7)&{}(2,1/2)(6,1/8)&{}(1,1)(8,1/9) \\ (3,1/4)(7,1/8)&{}(1,1)(9,1/9) &{}(1,1)(8,1/8) &{}(1,1)(9,1/9) &{}(2,1/3)(6,1/7)\\ (4,1/5)(6,1/7)&{}(3,1/4)(7,1/8)&{}(3,1/4)(5,1/6)&{}(5,1/5)(4,1/5)&{}(3,1/4)(5,1/6) \\ (5,1/6)(4,1/5)&{}(4,1/5)(5,1/6)&{}(4,1/5)(5,1/6)&{}(4,1/4)(5,1/5)&{}(4,1/5)(5,1/5) \\ (6,1/7)(3,1/3)&{}(7,1/8)(4,1/4)&{}(7,1/7)(2,1/3)&{}(6,1/7)(3,1/3)&{}(6,1/6)(3,1/3) \\ (5,1/6)(4,1/4)&{}(6,1/6)(3,1/4)&{}(4,1/6)(5,1/6)&{}(4,1/5)(6,1/6)&{}(5,1/7)(3,1/4)\\ (7,1/7)(2,1/5)&{}(6,1/7)(3,1/5)&{}(6,1/7)(2,1/5)&{}(5,1/6)(3,1/3)&{}(6,1/6)(4,1/5) \\ (4,1/5)(3,1/3)&{}(5,1/5)(2,1/4)&{}(3,1/5)(5,1/6)&{}(4,1/4)(5,1/6)&{}(5,1/5)(2,1/3)\\ \end{array} \right] \end{aligned},$$

50 $$\begin{aligned} B_{1}(A)=\left[ \begin{array}{lllll} (8,1/8)(1,1) &{}(9,1/9)(1,1) &{}(7,1/7)(2,1/2)&{}(8,1/8)(1,1) &{}(8,1/9)(1,1) \\ (7,1/7)(2,1/2)&{}(7,1/7)(3,1/3)&{}(3,1/9)(1,1) &{}(7,1/7)(2,1/2)&{}(7,1/9)(2,1/2) \\ (5,1/5)(4,1/4)&{}(6,1/6)(4,1/4)&{}(4,1/4)(5,1/5)&{}(5,1/5)(6,1/6)&{}(5,1/5)(3,1/3) \\ (4,1/4)(6,1/6)&{}(5,1/5)(6,1/6)&{}(5,1/6)(6,1/6)&{}(3,1/3)(7,1/7)&{}(3,1/4)(6,1/7)\\ (6,1/7)(3,1/3)&{}(3,1/3)(7,1/8)&{}(3,1/4)(5,1/7)&{}(5,1/5)(4,1/4)&{}(4,1/4)(5,1/6) \\ (3,1/3)(6,1/6)&{}(2,1/2)(6,1/6)&{}(1,1)(8,1/8) &{}(1,1)(8,1/8) &{}(2,1/2)(8,1/8) \\ (1,1)(8,1/9) &{}(1,1)(9,1/9) &{}(2,1/2)(7,1/7)&{}(2,1/3)(7,1/7)&{}(1,1)(9,1/9) \\ \end{array} \right] \end{aligned}.$$

Apply the proposed TOPSIS method to calculate the ration based on $d_{i}^{+}$ and $d_{i}^{-}$. One numeral example of the calculating results about $A_{1}$ and $B_{1}$ is summarized in Tables 7 and 8, where elements stands for $(d^{+},d^{-})$.

**Table 7:** The positive and negative distances of decision bodies *B* by $A_{1}$.

	$C_{1}$	$C_{2}$	$C_{3}$	$C_{4}$	$C_{5}$
$B_{1}$	(4.0000, 0.0000)	(4.0000, 0.0000)	(3.9464, 0.0000)	(4.0000, 0.0000)	(4.0000, 0.0000)
$B_{2}$	(1.2619, 3.7856)	(2.0000, 3.9464)	(1.8155, 3.7248)	(2.3928, 3.4723)	(1.2619, 3.7248)
$B_{3}$	(0.0000, 3.9464)	(1.2169, 3.7856)	(2.3928, 3.4723)	(0.0000, 3.6546)	(0.0000, 3.9464)
$B_{4}$	(2.3928, 3.7248)	(0.0000, 4.0000)	(0.0000, 3.7856)	(1.2169, 4.0000)	(1.8155, 3.4723)
$B_{5}$	(2.8284, 3.4723)	(2.3928, 3.7284)	(2.3928, 3.1789)	(1.6681, 2.8284)	(2.3928, 3.1789)
$B_{6}$	(3.1789, 2.8284)	(2.8284, 3.1789)	(2.8284, 3.1789)	(1.2619, 2.9222)	(2.8284, 2.9299)
$B_{7}$	(3.4723, 2.0000)	(3.7248, 2.5237)	(3.5425, 1.8155)	(2.2105, 2.0000)	(3.2619, 2.0000)
$B_{8}$	(3.1789, 2.5237)	(3.2619, 2.3928)	(3.0733, 3.1789)	(1.5665, 3.2169)	(3.3894, 2.3928)
$B_{9}$	(3.5425, 2.5129)	(3.4723, 2.6975)	(3.4723, 2.5129)	(1.9170, 2.0000)	(3.2619, 2.8284)
$B_{10}$	(2.8284, 2.0000)	(2.9299, 2.2083)	(2.6975, 3.1789)	(1.2619, 3.1789)	(2.9299, 1.8155)

**Table 8:** The positive and negative distances of decision bodies *A* by $B_{1}$.

	$C_{1}$	$C_{2}$	$C_{3}$	$C_{4}$	$C_{5}$
$A_{1}$	(3.7856, 0.0000)	(4.0000, 0.0000)	(3.5425, 1.2619)	(3.7856, 0.0000)	(3.9464, 0.0000)
$A_{2}$	(3.5425, 1.2619)	(3.5425, 2.0000)	(3.9464, 0.0000)	(3.5425, 1.2619)	(3.8856, 1.2619)
$A_{3}$	(2.9299, 2.5237)	(3.2619, 2.5237)	(2.5237, 2.9299)	(2.9299, 3.2619)	(2.9299, 2.0000)
$A_{4}$	(2.5237, 3.2619)	(2.9299, 3.2619)	(3.1789, 3.2619)	(2.0000, 3.5425)	(2.3928, 3.4723)
$A_{5}$	(3.4723, 2.0000)	(2.0000, 3.7248)	(2.3928, 3.3894)	(2.9299, 2.5237)	(2.5237, 3.1789)
$A_{6}$	(2.0000, 3.2619)	(1.2619, 3.2619)	(0.0000, 3.7856)	(0.0000, 3.7856)	(1.2619, 3.7856)
$A_{7}$	(0.0000, 3.9464)	(0.0000, 4.0000)	(1.2619, 3.5425)	(1.8155, 3.5425)	(0.0000, 4.0000)

### Sensitivity analysis

We apply a two-sided matching model to obtain the best pairs. To show the influence of decision results based on the best alternative and the worst alternative, we give the final matching results with different weights for the two kinds of weight vectors. All the results are summarized in Table 9. According to the matching results, we know that when the weights are $\mu =0.6$ and $\nu =0.4$, the error is the smallest and the matching result reaching the best, where the factor $\mu$ stands for the weight obtained by the Best-grade and the factor $\nu$ stands for the weight obtained by the Worst-grade, satisfying $\mu +\nu =1$.

**Table 9:** The matching results with different weights of Best-grade and Worst-grade.

Weight condition	Error	Matching result
$\mu =1$ ($\nu =0$)	3.1021	$(A_{1},B_{4}),(A_{2},B_{3}),(A_{3},B_{8}),(A_{4},B_{2}), (A_{5},B_{6}),(A_{6},B_{1}),(A_{7},B_{7})$
$\mu =0.9$ ($\nu =0.1$)	2.8490	$(A_{1},B_{4}),(A_{2},B_{3}),(A_{3},B_{8}),(A_{4},B_{2}), (A_{5},B_{6}),(A_{6},B_{1}),(A_{7},B_{7})$
$\mu =0.8$ ($\nu =0.2$)	2.6063	$(A_{1},B_{4}),(A_{2},B_{3}),(A_{3},B_{8}),(A_{4},B_{2}), (A_{5},B_{6}),(A_{6},B_{1}),(A_{7},B_{7})$
$\mu =0.7$ ($\nu =0.3$)	2.3634	$(A_{1},B_{4}),(A_{2},B_{3}),(A_{3},B_{8}),(A_{4},B_{2}), (A_{5},B_{6}),(A_{6},B_{1}),(A_{7},B_{7})$
$\mu =0.6$ ($\nu =0.4$)	2.1552	$(A_{1},B_{1}),(A_{2},B_{3}),(A_{3},B_{8}),(A_{4},B_{2}), (A_{5},B_{6}),(A_{6},B_{7}),(A_{7},B_{10})$
$\mu =0.5$ ($\nu =0.5$)	2.2645	$(A_{1},B_{4}),(A_{2},B_{9}),(A_{3},B_{7}),(A_{4},B_{4}), (A_{5},B_{3}),(A_{6},B_{5}),(A_{7},B_{10})$
$\mu =0.4$ ($\nu =0.6$)	2.5858	$(A_{1},B_{4}),(A_{2},B_{9}),(A_{3},B_{7}),(A_{4},B_{4}), (A_{5},B_{3}),(A_{6},B_{5}),(A_{7},B_{10})$
$\mu =0.3$ ($\nu =0.7$)	2.9067	$(A_{1},B_{4}),(A_{2},B_{9}),(A_{3},B_{7}),(A_{4},B_{4}), (A_{5},B_{3}),(A_{6},B_{5}),(A_{7},B_{10})$
$\mu =0.2$ ($\nu =0.8$)	3.2278	$(A_{1},B_{4}),(A_{2},B_{9}),(A_{3},B_{7}),(A_{4},B_{4}), (A_{5},B_{3}),(A_{6},B_{5}),(A_{7},B_{10})$
$\mu =0.1$ ($\nu =0.9$)	3.5489	$(A_{1},B_{4}),(A_{2},B_{9}),(A_{3},B_{7}),(A_{4},B_{4}), (A_{5},B_{3}),(A_{6},B_{5}),(A_{7},B_{10})$
$\mu =0$ ($\nu =1$)	3.8701	$(A_{1},B_{1}),(A_{2},B_{9}),(A_{3},B_{7}),(A_{4},B_{4}), (A_{5},B_{3}),(A_{6},B_{5}),(A_{7},B_{10})$

This case discusses a two-sided matching problem with two decision bodies’ attitudes. Each decision body expresses evaluation results under some important criteria. We apply the proposed two-sided matching model, obtaining the final matching result considering the two decision bodies’ satisfaction degrees. Because of different criteria weight values, the matching result may be different, leading to different decision results. As shown in Table 9, the matching results are partly different when the evaluations are made based on the best grade and worst grade. To elaborate slightly further on Table 9, the two-sided matching results are different when the weight factor $\mu$ based on the Best-grade and the weight factor $\nu$ are given different values. Introducing the concept of the weight coefficient, would increase the flexibility and practicability of the model. To analyze the impact of the weight coefficient objectively, we also introduce the concept of the error factor, which infers the best matching result. Table 9 shows that the smallest error occurs, when $\mu =0.6$ and $\nu =0.4$. It should be emphasized here that the values of the weight factor depends on the situations.

## Discussion

From the above analysis, the proposed two-sided matching method with I-BTM and LSGDM applied to high-level overseas talent and job fit problems takes full advantage of intuitionistic fuzzy sets, the BWM, TOPSIS and LSGDM. First, a hybrid bilateral matching method is constructed. Research on bilateral matching methods based on fuzzy sets mostly focuses on the aggregation of certain fuzzy evaluation information. We combine an intuitionistic multiplicative preference relationship with hesitant fuzzy language to express the evaluation information, which can better express the evaluator’s preference for the evaluation position and talent to be evaluated. Based on the BWM and TOPSIS, the two-sided matching method is expanded to enhance the scientific effectiveness of decision-making. For another, we developed an LSGDM method. At present, many studies on LSGDM are paying more attention to the consistency of decision-making information but ignoring the influence of social network relationships between decision makers on the evaluation results. Because the decision result is easily affected by the relationship between social networks in the person-job fit problem, we clustered the evaluators based on how close or distant the relationship is between the evaluators’ social networks. At the same time, we determined the evaluation criteria and the weights of the evaluators, although both considered the influence of the network to come from the inside and outside of the subgroup. Moreover, the proposed methods and models are efficient tools to handle the two-sided matching problem with LSGDM that take into account psychological behavior in the process of evaluation and a large number of DMs, which also provides an appropriate way to determine the criteria weights and evaluator weights in various evaluation or decision-making problems. In addition, we provide a reference for practice by building a mutual criteria system of overseas talent and jobs and showing the decision-making process combined with a case analysis.

## Conclusions and further research

In this paper, first, a novel two-sided decision-making model was constructed. In this process, we extended the BWM with intuitionistic preference relations, and the I-BWM was defined. To calculate the difference degree of the evaluating information, a novel distance measure was given. With this distance formula, the TOPSIS method was used to evaluate people and jobs. Second, we determined the weights of the evaluation standards and the evaluators by LSGDM considering the evaluators’ social networks. We analyzed the relations between the network nodes by calculating the node degree and eigenvector centralities and used a new algorithm to cluster them to obtain subgroups. Moreover, we took into account the influences of subgroups inside and outside. Finally, we established a mutual evaluation index set and introduced a general framework for solving two-sided matching decision problems such as the person-job fit procedure. The conclusion of the case study illustrated that the proposed method with I-BTM and LSGDM has some advantages. Although we have made some improvements in the research methods, there are still some limitations. This paper considers the social network relationship between evaluators, but we all know that the strength of the relationship between different people is not the same, and the degree of mutual influence between them is also not the same. The evaluation results of the decision makers will also be affected by the information they receive, which may change with time.

In future research, we will continue to pay attention to the process and status of overseas high-level talent. The matching relationship between overseas high-level talent and jobs is dynamic over time. Therefore, a time variable will be introduced in the matching process. Additionally, it is meaningful to analyze the impact of social network interaction, which occurs in the decision-making bodies of both sides. Due to the difference in the decision makers’ ability levels and experience, the incomplete information problem should also be considered.

## Abbreviations

BWM: Best-worst-method
DC: Degree centrality
DM: Decision maker
EC: Eigenvector centrality
I-BWM: Intuitionistic best worst method
IFN: Intuitionistic fuzzy number
LTS: Linguistic term set
LSGDM: Large-scale group decision making
MCDM: Multi-criteria decision making
OWA: Ordered weighted averaging
PLT: Probabilistic linguistic term
TOPSIS: Technique for Order of Preference by Similarity to Ideal Solution
